# Metagenomic complexity of high, seasonal transmission of *Plasmodium* spp. in asymptomatic carriers in Northern Sahelian Ghana

**DOI:** 10.1038/s43856-025-01088-y

**Published:** 2025-09-10

**Authors:** Mun Hua Tan, Oscar Bangre, Cecilia A. Rios-Teran, Kathryn E. Tiedje, Samantha L. Deed, Qi Zhan, Fathia Rasyidi, Mercedes Pascual, Patrick O. Ansah, Karen P. Day

**Affiliations:** 1 Department of Microbiology and Immunology, Bio21 Institute and The Peter Doherty Institute for Infection and Immunity, The University of Melbourne, Melbourne, VIC Australia; 2 Navrongo Health Research Centre, Ghana Health Service, Navrongo, Ghana; 3 Committee on Genetics, Genomics and Systems Biology, The University of Chicago, Chicago, IL USA; 4 Department of Biology, New York University, New York, NY USA

**Keywords:** Malaria, Parasitology, Metagenomics

## Abstract

**Background:**

Mixed-species, mixed-strain plasmodia infections are known to occur in humans in malaria endemic areas. It may be surprising that to date, the extent of this complexity has not been systematically explored in high-burden countries of sub-Saharan Africa, especially in the reservoir of asymptomatic infections in all ages, which sustains transmission.

**Methods:**

Here we take a metagenomic lens to these infections by sampling variable blood volumes from 188 afebrile residents living in high, seasonal transmission in Northern Sahelian Ghana. We estimated multiplicity of infection for different *Plasmodium* spp. through genotyping of antigens and microsatellites. We further defined ‘metagenomic complexity’ as a measure of overall within-host complexity across the combination of species and strains.

**Results:**

We show that prevalence of *Plasmodium* spp. and inter-/intra-species complexity is significantly higher in larger blood volumes from these individuals. Overall, malaria infections display high levels of metagenomic complexity comprising single-, double-, and triple-species infections with varying levels of intra-species complexity for *P. falciparum, P. malariae, P. ovale curtisi, and P. ovale wallikeri*. We also report a subset of individuals with highly-complex infections that cannot be explained by age or location. The implications of these findings to malaria epidemiology and control are illustrated by a geographic scaling exercise to district and region levels in northern Ghana.

**Conclusions:**

Our metagenomic investigation underscores the need to more sensitively measure within-host *Plasmodium* spp. complexity in asymptomatic carriers of infection. This will optimise strategies for malaria surveillance and control.

## Plain Language Summary

In malaria-endemic regions, people are chronically infected with parasites and may carry multiple species and strains at a time without symptoms. It is these infections that predominantly sustain transmission to mosquitoes. To improve malaria control and elimination strategies, better methods for detecting and measuring infections are needed. This study found that larger blood volumes detect more species and strain diversity, revealing that current approaches likely miss much of the infection complexity. We showed that some individuals carry more complexity than others, although the cause remains unknown. Our results highlight the limitations of current sampling practices and provide insights that could improve malaria surveillance in sub-Saharan Africa.

## Abstract (web summary)

Tan et al. apply a metagenomic lens to describe the *Plasmodium* species and intra-species complexity in asymptomatic carriers in a Northern Sahelian community in Ghana. This deep dive into the complexity of high transmission reveals large hidden reservoirs with potential to sustain transmission and challenge elimination in this region of Africa.

## Introduction

Hematozoan parasites represent a taxonomic group of eukaryotic microorganisms that infect a broad range of hosts. The most studied of this group are the causative agents of malaria, *Plasmodium* spp., a taxon that has been shown to be highly diverse^1^. The definitive hosts of malaria parasites, including humans, lizards, birds, and non-human primates, are frequently infected with multiple diverse species and strains (e.g., refs. ^2–8^). Despite this diversity, malaria is often too simplistically considered a disease caused by a single infectious agent, due in the case of humans to the dominating prevalence of a single species of *Plasmodium falciparum* that causes the major burden of infection globally. Often overlooked is the extreme genomic diversity of *P. falciparum* strains and other *Plasmodium* spp. in a host and how they may interact^5,9^.

The most recent World Health Organisation’s (WHO) World Malaria Report shows that 94% of malaria cases occur in sub-Saharan Africa (SSA), with most found in eleven high-burden countries in Africa, including Ghana^10^. In these high-transmission endemic areas, a large proportion of the population across all ages harbour the malaria parasites asymptomatically without clinical manifestation^11^. Repeated exposure to malaria infections drives the development of adaptive immune responses, shaping host immunity over time^12^. However, this immunity is often non-sterilising, protecting against clinical disease but not infection^13,14^ due to the extreme genomic diversity of *Plasmodium* spp., especially of *P. falciparum* with less known about the other species infecting humans (e.g., *Plasmodium vivax*, *Plasmodium malariae*, *Plasmodium ovale curtisi*, *Plasmodium ovale wallikeri*)^15–21^. This asymptomatic reservoir is sustained by enormous antigenic diversity of the parasite^22^, providing a vast infection reservoir for vector transmission^11,23,24^. The challenge of hyperendemic regions requires that the malaria field comes to terms with such diversity, studying it as a complex adaptive system to understand resilience to malaria interventions^25^.

Surveillance of the asymptomatic reservoir in humans has typically observed a large gradient in parasite density for *P. falciparum*, with most of these being submicroscopic or low-density infections with densities significantly lower than observed in clinical infections (Supplementary Fig. 1). Whilst it is known that individuals harbour infections with mixed species and strains^26,27^, the extent of this within-host complexity in the asymptomatic reservoir has never been explored. Here we present a metagenomic approach to study malaria in high transmission, given that malaria infection in a person comprises a community of parasites. Current reliance on whole genome sequencing with limitations in methods to phase genomes in highly-complex infections has forced the field to view infections simplistically by examining dominant parasites whilst ignoring rare species or strains with potential for emergence. In contrast, a key aspect of metagenomic experimental design is ensuring deep sampling to capture most of the complexity of a community, as employed in environments as diverse as human microbiome and environmental microbiology^28–30^. Similarly, this must be considered when designing malaria surveillance and genetic studies in high transmission, where the end game is to eliminate the species or detect the emergence of drug-resistant parasites. It is currently unknown if the routine sampling of a small blood volume from dried blood spot (DBS) cuttings in epidemiological surveys is reflective of actual metagenomic complexity per person in high transmission.

We set out to explore the impact of sampling blood volume on the metagenomic complexity of *Plasmodium* spp. in 188 afebrile human hosts in four age groups, living in high transmission in Bongo District in the Upper East Region of northern Ghana. We report significantly higher prevalence of *Plasmodium* spp. with large volume sampling at 100 μL packed red blood cells (pRBC) compared to DBS, and also observe higher *P. falciparum* complexity defined as multiplicity of infection (MOI). This contributes to increased richness in the parasite population whilst low genetic similarity among isolates is maintained. We show that complexity of malaria infections in Northern Sahelian Ghana^31^ comprises single-, double-, and triple-species infections with varying MOI levels per species and identify a subset of individuals with highly-complex infections. Our findings determine that broad sampling across a population (i.e., sampling many individuals) and deep sampling within individuals (i.e., sampling large blood volumes) in a reservoir is crucial for accurate representation of the parasite population in an endemic area, requiring us to view complexity cumulatively in a metagenomic sense, not only of the dominant *P. falciparum* species but also of the minor *Plasmodium* spp. inclusively. These findings are of translational significance to develop sentinel site protocols relevant to local epidemiological conditions in high-transmission areas of West Africa moving towards pre-elimination.

## Methods

### Inclusion and ethics

This study was reviewed and approved by the ethics committees at the Navrongo Health Research Centre (Ghana; NHRC IRB-131), The University of Melbourne (Australia; Project IDs: 13433, 31586, and 21649), The University of Chicago (USA; IRB14-1495, IRB19-0760, and IRB21-0417), and New York University (USA; IRB-FY2024-8572). Details on the study area, study population, inclusion/exclusion criteria, and data collection procedures have been described in refs. ^32,33^. Informed consent was obtained from key stakeholders and the local community in Bongo District before this research was undertaken. Members of the local community were trained as field workers and were directly involved in liaising with the community and in the collection of the study data.

### Study area and population

This study was conducted in the Bongo District in the Upper East Region of northern Ghana, characterised by seasonal malaria transmission, with a short, wet season (Jun-Oct) and a prolonged dry season (Nov-May)^32,33^. For this study, supplementary consent was sought from 200 enrolled individuals in the Vea/Gowrie catchment area to collect ~5 mL of whole blood. For this collection, 50 individuals were randomly chosen from four age groups (i.e., 6–10, 11–20, 21–39, ≥40 years). This was completed during the larger cross-sectional study in Bongo that enrolled ~2000 individuals in November 2020. At the time of this survey, the population had undergone malaria control interventions, including long-lasting insecticidal nets (LLINs), three rounds of indoor residual spraying (IRS) (2013–2015), and five consecutive years (2016–2020) of seasonal malaria chemoprevention (SMC) administered at monthly intervals during the malaria season to children between the ages of 3–59 months (i.e., <5 years old)^22^.

### Estimation of WB and pRBC volume equivalent in DBS

The typical protocol in the field involves collecting 3–4 dried blood spots (DBS) per filter paper^34^. From these small DBS, two sections are cut ( ~ 5 mm × 5 mm each) for gDNA extraction. We approximated that two 5 mm × 5 mm DBS cuttings contain the equivalent of ~6–7 μL of whole blood (Supplementary Method. 1). Given the average expected proportion of RBCs at ~40%, our typical DBS cuttings would thus have the equivalent of ~2.4–2.8 μL packed red blood cells (pRBC), with some variation due to a person’s hermatocrit levels. Thus, this study set out to compare MOIs estimated using gDNA extracted from pRBC volumes of 1 μL, 10 μL, 50 μL, and 100 μL.

### Parasitological measurements

Parasitological measurement methods were performed according to Tiedje et al.^32^. Parasite densities were counted against 200 white blood cells (WBC) on 10% Giemsa-stained thick film blood smears and examined under oil immersion of 100-fold magnification. Final parasite densities (parasites/mL of blood) were calculated by averaging two independent readings, assuming an average WBC count of 8000/μL of blood. Parasite species were identified using a 100-fold magnification of thin film smears and categorised based on morphology.

### Sampling of venous whole blood and dried blood spot samples

Two hundred individuals with ages ranging 6–90 years were sampled. Children <5 years receiving SMC were excluded. Approximately 5 mL of venous whole blood samples (WB) were collected per person and transported to the laboratory at the Navrongo Health Research Centre in Ghana, where they were processed within 2–4 h after collection. These WB samples were centrifuged at 3000 RPM at 4 °C for 10 min to separate the WB into components of pRBC, WBC, and plasma. All three components were harvested individually and stored at −80 °C.

At the same time as the venous whole blood collection, DBS were also collected onto filter paper (3MM Whatman) for the same individuals and for a larger subset of the population in Bongo (see Tiedje et al.^22^).

### Extraction of genomic DNA from pRBC and DBS

Of the 200 individuals, a total of 12 were excluded (Supplementary Table. 1). Nine were excluded as these could not be matched accurately to epidemiological data. The remaining three individuals were excluded as these were later confirmed to be symptomatic (i.e. febrile and microscopically-positive for *P. falciparum*). Genomic DNA (gDNA) was extracted from four volumes of pRBC (1 μL, 10 μL, 50 μL, 100 μL) from parasites ‘isolates’ of the remaining 188 afebrile individuals, using the QIAamp DNA Blood Mini Kit, following manufacturer’s protocol (Cat No. 51106). A 1× PBS was added to pRBC samples to make up 200 μL of every sample volume for extraction. An RNase A step was included to generate RNA-free genomic DNA, following manufacturer’s protocol (Cat No. 19101). Genomic DNA was eluted in 50 μL buffer AE. In this study, a ‘sample’ refers to a DBS or pRBC volume per isolate, hence there can be multiple samples per isolate. These extractions were performed in 2022 (August to September).

Two 5 mm × 5 mm sections were cut from DBS and gDNA extraction was performed using the QIAamp DNA mini kit (QIAGEN) according to manufacturer’s protocol with modifications^32^. Genomic DNA was eluted in 50 μL buffer AE. These extractions were performed in 2022 (March to June).

### Measurement of plasma PfHRP2 concentrations

Plasma PfHRP2 concentration was determined using the Quantimal CELISA kit (TM, Cellabs). In this enzyme-linked immunosorbent assay, wells are coated with a primary monoclonal antibody to PfHRP2, sample was applied and wells were washed, then probed with a secondary anti-Pf antibody conjugated to horseradish peroxidase (HRP) for detection using the FLUOstar omega plate reader (BMG Labtech) at 450 nm. A standard curve was prepared starting at 10 ng/mL of standardised PfHRP2 and diluted 1:2 down to 0.01 ng/mL. The negative control of Melbourne plasma (Australian Red Cross Lifeblood, non-malarious region) was included for comparison. Individual plasma samples were collected as mentioned above, and diluted 1:20 in duplicate, along with the negative control and RPMI blanks, to allow most positive samples to fall within the range of detection of the assay. Where the sample was saturated it was repeated at 1:40, and where it was detected but fell under the cut-off it was repeated at 1:10 to confirm results. All standard curves and samples were analysed using Arigo GAINdata ELISA data analysis software (Arigo biolaboratories) to obtain PfHRP2 concentrations, and the cut-off was applied manually at the level suggested by the manufacturer (OD negative control+0.1, Cellabs).

### Detection of *Plasmodium* spp. using the species-specific *18S rRNA* PCR

For all the DBS and 100μL-pRBC samples (*N* = 188 isolates) a nested PCR targeting the 18S ribosomal RNA gene (*18S rRNA*) of *Plasmodium* spp. was performed using previously published primers and protocols with modification^32,35^to detect for the presence of *P. falciparum, P. malariae, P. ovale* spp., and/or *P. vivax* in all samples^32,35^. The protocol and primer sequences for the species-specific *18S rRNA* PCR are available online on GitHub (see Data Availability).

Both the first and second rounds of the nested PCR were carried out in a total volume of 20 μL, with final concentrations of 1X buffer, 2 mM of MgCl_2_, 0.125 mM of dNTP mix, 0.125 μM of each primer, 0.4 units of GoTaq G2 Flexi DNA polymerase (Promega), and nuclease-free water. The first round was carried out using 2 μL of gDNA template and the rPLU6/rPLU5 primer pair. The second round was carried out in four separate tubes, each containing a single species-specific primer pair (i.e., rFAL1/rFAL2 to detect the presence of *P. falciparum*, rMAL1/rMAL2 for *P. malariae*, rOVA1/rOVA2 for *P. ovale* spp., rVIV1/rVIV2 for *P. vivax*) and 2 μL of the first-round PCR product. The PCR amplification was performed in two rounds with the following cycling conditions: the first round consisted of 95 °C for 2 min; 25 cycles of 58 °C for 2 min, 72 °C for 5 min, and 94 °C for 1 min; followed by 58 °C for 2 min, 72 °C for 2 min, and storage at 4 °C. The second round consisted of 95 °C for 2 min; 30 cycles of 58 °C for 2 min, 72 °C for 5 min, and 94 °C for 1 min; followed by 58 °C for 2 min, 72 °C for 2 min, and storage at 4 °C. For each PCR, positive (i.e., *P. falciparum* 3D7 isolate) and negative (i.e., nuclease-free water) controls were included for quality control. The PCR amplicon products from the four separate second-round PCRs (i.e., *P. falciparum, P. malariae, P. ovale* spp., *P. vivax*) (10 µL) were electrophoresed for 90 min on a 2% agarose gel stained with SYBR^TM^ Safe DNA Gel Stain (Invitrogen) along with a 100 bp DNA Ladder (100 bp to 3,000 bp) (Axygen). The different *Plasmodium* spp. were determined to be present in each isolate based on band size (*P. falciparum*: 205 bp, *P. malariae*: 144 bp, *P. ovale* spp.: 800 bp, *P. vivax*: 120 bp).

### Genotyping of *P. malariae* using microsatellites

Of the 188 isolates, 26 were identified as *P. malariae*-infected isolates using the species-specific *18S rRNA* PCR. The 100 µL-pRBC sample for each of these isolates was genotyped at 12 neutral microsatellite loci using primers from two previously published panels^36,37^. For this study, the two panels were combined and multiplexed. Outer primers were designed for the first round of amplification in the Mathema et al.^37^ panel to enable a semi-nested PCR approach, while the Bruce et al.^36^ primers were used as published. The protocol and primer sequences are available online on GitHub (see Data Availability).

The PCR protocol and cycling conditions were optimised to maximise amplification success, particularly for samples with low parasite densities. The first-round PCRs were multiplexed (i.e., three separate PCR reactions each containing four different sets of primers). Each first-round of this semi-nested PCR was carried out in a total volume of 25 µL, comprising 5 µL of gDNA template, with final concentrations of 1X buffer, 3 mM of MgCl_2_, 0.4 mM of dNTP mix, 0.04 µM of each primer, 1.2 units of GoTaq G2 Hot Start polymerase (Promega), and nuclease-free water. The second-round PCRs required the preparation of 12 separate reactions with the fluorescent-labelled primers. Each second-round PCR was carried out in a total volume of 25 µL, comprising 3 µL of the first-round PCR product, with final concentrations of 1X buffer, 3 mM of MgCl_2_, 0.2 mM of dNTP mix, 0.08 µM of each primer, 0.6 units of GoTaq G2 Hot Start polymerase (Promega), and nuclease-free water. The PCR amplification was performed in two rounds with the following cycling conditions: the first round consisted of 94 °C for 4 min; 35 cycles of 94 °C for 30 sec, 48 °C for 30 sec, and 68 °C for 1 min; followed by 68 °C for 2 min and storage at 4 °C. The second round consisted of 94 °C for 4 min; 45 cycles of 94 °C for 30 sec, 52 °C for 30 sec, and 68 °C for 1 min; followed by 68 °C for 2 min and storage at 4 °C. For each PCR, positive (i.e., previously genotyped *P. malariae* isolates) and negative (i.e., nuclease-free water) controls were included for quality control.

For the capillary electrophoresis, four pools with the fluorescently labelled round-two PCR products were prepared for each *P. malariae* isolate (available online on GitHub). These pools were then sent to the Australian Genome Research Facility (AGRF, Melbourne, AU) where capillary electrophoresis was performed on an Applied Biosystems 3730xl DNA analyser with a 50 m array and POP-7 polymer (ThermoFisher Scientific). Analysis of the raw electropherograms was carried out using GeneMarker® software v3.0.1 (SoftGenetics LLC) and alleles were scored using customised panels and normalised to the size standard LIZ500. To prevent the mis-scoring of stutter peaks, multiple alleles per locus were scored if the minor peak(s) height was > 33% of the predominant allele or highest peak (i.e., local max) in the electropherogram. Background noise was defined as any peak < 200 fluorescent units^38^. For all isolates genotyped, the multilocus haplotypes were constructed using the predominant allele (or highest peak) at each locus. Automated binning was performed using TANDEM v. 1.07^39^.

### Identification and genotyping of *P. ovale* spp. using *potra*

Since its identification by Stephens in 1922, *Plasmodium ovale* was considered a single species. However, molecular studies later revealed two genetically distinct forms, commonly referred to as *P. ovale curtisi* and *P. ovale wallikeri*^20^. While the formal nomenclature of these species remains under discussion^21,40–42^, this study adopts the prevailing convention in the literature, referring to the two species as *P. ovale curtisi* and *P. ovale wallikeri*, and using *P. ovale* spp. to describe them collectively.

Of the 188 isolates, 10 were identified as *P. ovale* spp.-infected isolates using the species-specific *18S rRNA* PCR. The 100 µL-pRBC sample for each of these isolates was genotyped for *P. ovale* spp. identification (i.e., *P. ovale curtisi* or *P. ovale wallikeri*) and genetic diversity. This semi-nested PCR protocol involves species-specific amplification of a size-polymorphic fragment of the single-copy *P. ovale* spp. tryptophan-rich antigen gene (*potra*)^20,43,44^ with modifications for low-density infections. The method was also used because it specifically targets the species-specific *potra* regions (i.e., *poctra* for *P. ovale curtisi* and *powtra* for *P. ovale wallikeri*) enabling both species discrimination and intra-species genetic diversity based on the PCR amplicon size. The protocol and primer sequences for *potra* are available online on GitHub (see Data Availability).

The first-round of this semi-nested PCR was carried out in a total volume of 20 µL, comprising 2 µL of gDNA template, with final concentrations of 1X buffer, 2 mM of MgCl_2_, 0.125 mM of dNTP mix, 0.125 µM of each primer (PoTRA-F/PoTRA rev3), 0.4 units of GoTaq G2 Flexi DNA polymerase (Promega), and nuclease-free water. The second-round PCRs involved two separate reactions using the forward primer (PoTRA-F) and either the *P. ovale curtisi* (PocTRA-R) or the *P. ovale wallikeri* (PowTRA-R) reverse primer. Each second-round PCR was carried out in a total volume of 20 µL, comprising 3 µL of the first-round PCR product, and the same final reagent concentrations as the first round. The PCR amplification was performed in two rounds with the following cycling conditions: the first round consisted of 95 °C for 4 min; 25 cycles of 95 °C for 1 min, 56 °C for 1 min, and 72 °C of 1 min; followed by 72 °C for 2 min and storage at 4 °C. The second round consisted of 95 °C for 4 min; 30 cycles of 95 °C for 1 min, 60 °C for 1 min, and 72 °C for 1 min; followed by 72 °C for 2 min and storage at 4 °C.

The *P. ovale curtisi* and/or *P. ovale wallikeri* PCR amplicon products from the second round (10 µL) were electrophoresed for 120 min on a 2% agarose gel stained with SYBR^TM^ Safe DNA Gel Stain (Invitrogen) along with a 100 bp DNA Ladder (100 bp to 3000 bp) (Axygen). To estimate the size (bp) of the *P. ovale curtisi and/*or *P. ovale wallikeri* PCR amplicon(s) identified for each isolate, a point-to-point regression method implemented in the BIORAD Image Lab Software V5.2.1 (installed on the BIORAD Gel Doc^TM^ EZ Imager) was used. The point-to-point regression method (semi-log) estimates the molecular weight by interpolating the distances of the PCR amplicons to those of the 100 bp DNA Ladder. The bins were constructed every 18 bp which corresponds to the length of the repeat size of the amplified *poctra* and *powtra* fragments. Automated binning was performed for *poctra* and *powtra* using TANDEM v. 1.07^39^, recorded in Microsoft Excel v. 2211, and analysed in RStudio version 4.2.2.

### Targeted amplicon sequencing of *P. falciparum* DBLα tags with *var*coding

The *var*coding method has been shown to be appropriate for MOI estimation in high transmission, outperforming SNP-based barcodes^45,46^. This method utilises targeted amplicon sequencing of *var* DBLα type sequences encoding a Duffy-binding-like domain of *Plasmodium falciparum* erythrocyte membrane protein 1 (PfEMP1). For DBS samples, *var*coding was performed on microscopy-positive isolates using published primers and protocols^22^.

For pRBC samples, a modified protocol was applied. *Var*coding was performed on four pRBC volumes for 188 isolates, including those that appear negative by microscopy or species-specific PCR to assess if more infections are found when larger volumes are sampled. Forty isolates (i.e. ~20% of isolates) were randomly selected as repeats to assess reproducibility. Laboratory strains (i.e., 3D7, Dd2, HB3) were included as positive controls. Samples of varying pRBC volumes of a same isolate and its repeats (if any) were sequenced on the Illumina platform using the MiSeq Reagent Kit v3 (600 cycle; 2 × 300 bp paired-end) in the same sequencing pool and run.

The sequence region within *var* genes encoding the DBLα domain of PfEMP1 (i.e. DBLα tags) were amplified in a single-step PCR from genomic DNA using universal degenerate primer sequences^22^ to blocks D (forward primer: DBLαAF, 5’-GCACGMAGTTTYGC-3’) and H (reverse primer: DBLαBR, 5’-GCCCATTCSTCGAACCA-3’)^47,48^. To generate data in this study, primers used included forward barcoded primers and an unbarcoded reverse primer. Both forward and reverse primers also contain Illumina Nextera overhangs to facilitate subsequent preparation of sequencing libraries. Primer sequences are available online on GitHub (see Data Availability).

Each PCR was prepared in a total volume of 40 μL, comprising of 2 µL of gDNA template, with final concentrations of 0.5X buffer, 2 mM of MgCl_2_, 0.07 mM of dNTP mix, 0.375 μM of each primer (DBLαAF, DBLαBR), 3 units of GoTaq G2 Flexi DNA polymerase (Promega), and nuclease-free water. The PCR cycling conditions consisted of 95 °C for 2 min; 30 cycles of 95 °C for 40 sec, 49 °C for 90 sec, and 65 °C for 90 sec; followed by at 65 °C for 10 min and storage at 4 °C. PCR products were purified using the SPRI method (solid-phase reversible immobilization) (Agencourt AMPure XP beads). Purified PCR product concentrations were measured using the Quant-iT PicoGreen dsDNA Kit (Invitrogen, Cat No. P7589) following manufacturer’s protocol. Amplicons were then pooled equimolarly, with each pool consisting of up to 99 isolates, all with unique barcodes. Pooled amplicons were subsequently indexed (Amplicon Indexing Service) and sequenced on the Illumina platform using the MiSeq Reagent Kit v3 (600 cycle; 2 × 300 bp paired-end) at the Australian Genome Research Facility (AGRF, Melbourne, AU).

### Processing DBLα tags into DBLα types

An established bioinformatics workflow^49,50^ consisting of a suite of pipelines was used to generate DBLα tags from raw Illumina paired-end reads (https://github.com/UniMelb-Day-Lab/tutorialDBLalpha). DBLα tag sequence data included DBLα tags from pRBC generated in this study, combined with DBLα tags from an interrupted time-series study in Bongo, involving one pilot and eight cross-sectional surveys conducted between 2012 and 2020 (i.e., Malaria Reservoir Study, Genbank BioProject PRJNA396962). These surveys involved mostly asymptomatic infections^22,32,51^ with small proportions of symptomatic and clinical infections. Clustering of DBLα tags was performed at a 96% nucleotide identity threshold^52^ used to define a DBLα tag in a sample or DBLα types in a population of tags.

- The DBLaCleaner pipeline (v1.0)^49^ was used to generate DBLα tag sequences per sample from raw paired-end Illumina sequence reads.
- The clusterDBLa pipeline (v1.0)^49^ was used to generate unique DBLα types from DBLα tags with a matrix detailing presence/absence of each DBLα type in isolates.
- The classifyDBLa pipeline (v1.0)^50^ was used to classify DBLα types into DBLα domain subclasses and subsequently into upsA or non-upsA groups^53^.

### Estimation of isolate repertoire size and multiplicity of infection (MOI)

The isolate repertoire size represents the number of unique DBLα types in an isolate. Pf-MOI represents the estimated number of unique parasite genomes in an isolate. For samples with ≥20 DBLα types, Pf-MOI in each sample was estimated using a Bayesian approach (prior = uniform, aggregate = pool)^22^. Samples with ≥1 DBLα types but <20 DBLα types were assigned Pf-MOI = 1. *Plasmodium malariae* MOI (Pm-MOI) was estimated based on the maximum number of distinct alleles at any of the 12 microsatellite loci. *Plasmodium ovale* spp. MOI (Po-MOI) was estimated from the number of unique fragment sizes obtained based on the *potra* gene.

### Estimation of fold difference (FD_*Pf-MOI*_) and model fitting

Differences in Pf-MOI were expressed as fold differences (FD):

1 $${{FD}}_{{MOI}}=\frac{{{MOI}}_{L}}{{{MOI}}_{S}}$$

where MOI_*S*_ and MOI_*L*_ are the Pf-MOI of the smaller (S) and larger (L) volume samplings, respectively, of a same isolate. A generalised additive model (GAM) was fit to the data (FD_*Pf-MOI*_ and Pf-MOI_*S*_) with penalised smoothing parameters selected by REML ( *gam* function in mgcv v1.9-1^54^).

### Estimation of genetic similarity or overlap between isolate repertoires

To ensure robust analyses exploring genetic similarity, we evaluated only samples with ≥ 20 DBLα types (i.e. isolate repertoire size ≥ 20). Genetic similarity of repertoires of two isolate repertoires is generally represented by the pairwise type sharing metric (PTS)^52^. Specifically:

2 $${PTS}=\frac{2* {{shared}}_{{ij}}}{{{Size}}_{i}+{{Size}}_{j}}$$

where shared_*ij*_ is the shared number of DBLα types between isolate repertoires of samples *i* and *j*, and Size_*i*_ and Size_*j*_ are the isolate repertoire sizes of samples *i* and *j*, respectively. A value of 0 indicates the absence of sharing between two samples while a value of 1 indicates completely identical DBLα repertoires.

Genetic similarity between isolate repertoires from two different pRBC volumes was assessed in a directional manner relative to the isolate repertoire size of a reference sampled volume (i.e. denominator)^49^:

3 $${{PTS}}_{S}=\frac{{{shared}}_{{SL}}}{{{Size}}_{S}};{{PTS}}_{L}=\frac{{{shared}}_{{SL}}}{{{Size}}_{L}}$$

where shared_*SL*_ is the shared number of DBLα types between isolate repertoires of smaller (S) and larger (L) volume samplings of a same isolate, and Size_*S*_ and Size_*L*_ are the isolate repertoire sizes of smaller and larger volume samplings, respectively, of a same isolate (i.e., Vol_*S*_ and Vol_*L*_). High PTS_*S*_ and low PTS_*L*_ values indicate that DBLα types identified in the smaller volume are also present in the larger volume and that there are additional DBLα types identified in the larger volume but not found in the smaller volume.

### Scaling up prevalence, Pf-MOI, and *P. falciparum* census population size to larger regions in Bongo and Upper East Ghana

Prevalence, mean MOI, and *P. falciparum* census population size^22^ values were estimated from DBS samples of a larger surveyed 1455 individuals aged ≥6 years (Supplementary Method. 2). These values were further adjusted to obtain estimates if isolates were sampled more deeply at 100 μL (Supplementary Data. 1), using non-linear correlations comparing Pf-MOI from 1 μL and 100 μL pRBC. Further scaling up to the larger population sizes of Bongo District and Upper East Region of Ghana provided an approximation of the number of infections or parasites underestimated in these regions. Given the exclusion of children ≤ 5 years from this study, population size data was split for 0–9 years with a 6:4 ratio into two age groups 0–5 years and 6–9 years. Relevant to our study. The final age-structured population size data as follows:

- Bongo District: 102,004 [ ≥ 6 years]; 12,166 [6–9 years]; 28,494 [10–19 years]; 35,135 [20–39 years]; 26,209 [ ≥ 40 years].
- Upper East Region: 1,100,676 [ ≥ 6 years]; 133,700 [6–9 years]; 304,105 [10–19 years]; 378,958 [20–39 years]; 283,913 [ ≥ 40 years].

Map of regions and districts in Ghana was drawn with Global ADMinistrative area (GADM) data downloaded for Ghana (v4.1)^55^. From the Malaria Atlas Project (MAP)^56,57^, we downloaded data on ‘Infection Prevalence’ standardised based on the proportion of children aged 2–10 years in Ghana in 2020 (also the year of our field sampling). Infection prevalence in children aged 2–10 years in Bongo is shown (pixel location: ‘Bongo, Upper East, Ghana’).

### Statistical analysis

Differences in the number of *Plasmodium* spp. detected between DBS and 100µL-pRBC were analysed with Fisher’s exact test (two-sided, *stats* v4.2.1^58^). The Shapiro-Wilk test of normality rejected normality of Pf-MOI data. The nonparametric Friedman test (*rstatix* v0.7.2^59^) compared MOI distributions for paired data of four pRBC volumes. Nonparametric pairwise Wilcoxon signed rank tests (two-sided) with “holm” correction (*rstatix* v0.7.2^59^) compared MOI data of paired pRBC volumes or repeats. Lin’s concordance correlation coefficient (CCC) (*DescTools* v0.99.49^60^) estimated the agreement of MOI values between pRBC volumes for a same isolate or between two repeats of a same isolate. The Kruskal-Wallis Rank Sum test (*rstatix* v0.7.2^59^) compared PTS distributions between repeats for each pRBC volumes, with Dunn’s test (*rstatix* v0.7.2^59^) for multiple comparisons. Association of variables with metagenomic complexity was tested using the two-sided Mann-Whitney U test (sex, village), the Kruskal-Wallis test (occupation, section), and Spearman’s correlation (age, haemoglobin, temperature). Tests with *p-value* or adjusted *p*-value ≤ 0.05 were considered significant.

To obtain age-specific correction scales, we must have sufficient data with MOI > 1 in each pRBC volume and/or host age groups. To determine the minimum sample sizes to achieve enough statistical power, we first calculated effect sizes (h) from the proportion of isolates with MOI > 1 using the *ES.h* function in the *pwr* R package (v1.3-0)^61^. The *pwr.2p2n.test* function from the same R package was used to estimate statistical power at a significance level (α) of 0.05 (alternative = “two.sided”). The *pwr.2p.test* function was further used to estimate the minimum sample sizes (n) required to achieve a statistical power of 0.80 (α = 0.05, alternative = “two.sided”) for future correction by host age groups.

### Reporting summary

Further information on research design is available in the Nature Portfolio Reporting Summary linked to this article.

## Results

**Fig. 1:**
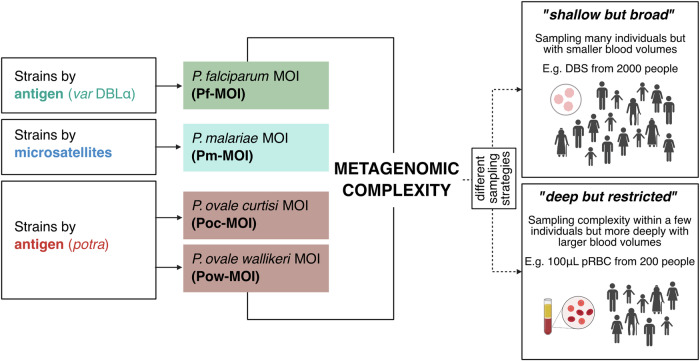
Defining terminologies in this study. We define ‘strains’ of malaria parasites as genetically-diverse parasites that are distinct either antigenically (for *P. falciparum* and *P. ovale* spp.) or by genotypes based on neutral microsatellites (for *P. malariae*). Multiplicity of infection (MOI) represents the number of different strains per person. Metagenomic complexity summarises MOI across the combination of species as a measure of overall within-host complexity. We also define the different sampling strategies discussed in this study: ‘shallow but broad sampling’ referring to sampling many individuals but with smaller blood volumes (e.g., dried blood spots (DBS)), as opposed to ‘deep but restricted sampling’ when referring to sampling MOI or complexity within a few individuals but with larger blood volumes (e.g., 100 μL packed red blood cells (pRBC)). Created in https://BioRender.com.

To measure infection complexity across species, we define here terms used in this study (Fig. 1). Sex is an obligatory part of the *Plasmodium* spp. life-cycle and constant outcrossing occurs in nature, especially in high transmission^62,63^. Therefore, unlike in bacteriology where strains are defined as clonal entities, this study defines ‘strains’ as genetically-diverse parasites that are distinct either by antigen (for *P. falciparum* and *P. ovale* spp.) or by neutral markers (microsatellites, for *P. malariae*). The number of different strains per person is represented by ‘multiplicity of infection’ (MOI). ‘Metagenomic complexity’ further summarises MOI across the combination of species as a measure of overall within-host complexity.

### Demography of surveyed individuals and parasitological characteristics of infections

This study was conducted in the Bongo District (Vea/Gowrie catchment area) in the Upper East Region of northern Ghana, characterised by seasonal malaria transmission^32,33^. Based on the WHO “A Framework for Malaria Elimination”^64^, this area is categorised as high transmission where *P. falciparum* microscopic prevalence was ≥35% at baseline in 2012^32,33^. A study population of 188 individuals (i.e., 100 female and 88 male based on self-reporting) with age ranges 6–90 years were sampled randomly at the end of the wet season in November 2020 (Supplementary. Tables 1-2). Children <5 years were receiving seasonal malaria chemoprevention (SMC) and excluded. Of the 188 individuals, 47 (25.0%) were adolescents (11–20 years) and 95 (50.5%) were adults ( ≥ 21 years), generally excluded in current malaria surveillance where the target is measuring disease burden^65^. Parasite ‘isolates’ from only 33 of the 188 individuals (17.6%) were found to be microscopically-positive for *Plasmodium* spp., predominantly of *P. falciparum*, including mixed infections. We also identified 46 microscopy-negative isolates with positive *P. falciparum* histidine-rich protein 2 (PfHRP2) measurements (Supplementary Table 3).

### Higher prevalence of *Plasmodium* spp. multi-species infections in larger pRBC volumes

**Fig. 2:**
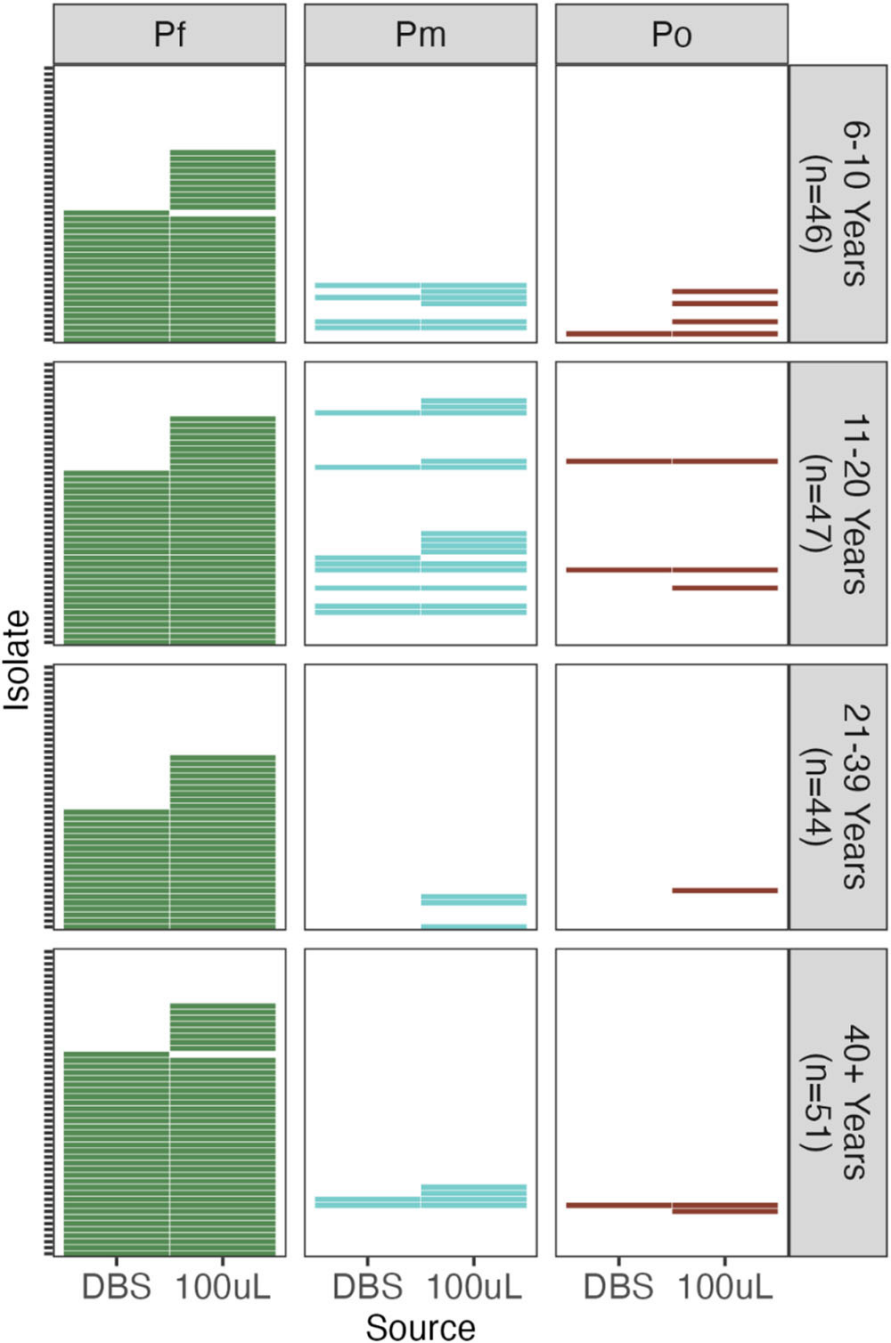
Relative to sampling of DBS, sampling of larger pRBC volumes yielded increased prevalence of *Plasmodium* spp. infections. Data shown for *Plasmodium* spp. detection by species-specific *18S rRNA* PCR on DBS and 100μL-pRBC for *N* = 188 isolates, stratified by host age (6–10 years (*n* = 46 isolates), 11–20 years (*n* = 47 isolates), 21-39 years (*n* = 44 isolates), and ≥40 years (*n* = 51 isolates)). All infections were negative for *P. vivax* and therefore excluded from this figure. Presence or absence of *P. falciparum*, *P. malariae*, and *P. ovale* spp. as indicated by Pf, Pm, and Po respectively.

Overall, *Plasmodium* spp. prevalence was underestimated when DBS cuttings or small blood volumes (1 μL) were sampled. Based on *18S rRNA*, parasite detection levels were significantly higher in 100 μL packed red blood cells (pRBC) relative to those in DBS, by factors of 1.32× for *P. falciparum*, 1.93× for *P. malariae*, and 2.50× for *P. ovale* spp. (Fig. 2, Table 1). An independent *var*coding protocol corroborated *P. falciparum* findings, indicating 1.36× detection in 100 μL compared to 1 μL-pRBC (Supplementary Table. 4). *P. vivax* remained undetected in all infections regardless of blood volume sampling. Increased prevalence of double- and triple-species infections in 100 μL-pRBC relative to DBS was also observed (Fig. 2). Parasite prevalence relies on counting the proportion of human hosts infected but does not capture within-host parasite population sizes; we explore this within-host complexity and diversity in the subsequent sections.

**Table 1:** Number of detected infections by species and age group

Number of detected infections by species & age group						
Species	Host Age Group	Microscopy positive^a^	DBS^b^	100 μL pRBC^b^	Sig.	× increase^c^
*P. falciparum*	All	33	105	139	****	1.32
	6–10 Years	9	22	31	***	1.41
	11–20 Years	12	29	38	****	1.31
	21–39 Years	4	20	29	****	1.45
	≥40 Years	8	34	41	****	1.21
*P. malariae*	All	2	14	27	****	1.93
	6–10 Years	0	4	6	****	1.50
	11–20 Years	2	8	14	***	1.75
	21–39 Years	0	0	3	ns	4.00^c^
	≥40 Years	0	2	4	**	2.00
*P. ovale* spp.	All	0	4	10	****	2.50
	6–10 Years	0	1	4	ns	4.00
	11–20 Years	0	2	3	**	1.50
	21–39 Years	0	0	1	ns	2.00^c^
	≥40 Years	0	1	2	*	2.00

Data shown for *Plasmodium* spp. detection by species-specific *18S rRNA* PCR on DBS and 100 μL-pRBC for *N* = 188 isolates, stratified by host age (6-10 years (*n* = 46 isolates), 11–20 years (*n* = 47 isolates), 21–39 years (*n* = 44 isolates), and ≥40 years (*n* = 51 isolates)). A two-sided Fisher’s exact test was used to assess significance in differences between DBS and 100uL-pRBC, with symbols *, **, ***, and **** representing *p*-value significance levels of 0.05, 0.01, 0.001, and 0.0001, respectively.

^a^This column represents the number of individuals sampled with isolates that were microscopically-positive for *Plasmodium* spp., predominantly of *P. falciparum*, including mixed infections.

^b^These columns represent the number of individuals sampled with isolates that were *18S rRNA* PCR positive for *Plasmodium* spp., including mixed infections.

^c^For some groups (marked), due to zero values for DBS, this was calculated with the addition of 1 to each value. E.g. for *P. malariae*, 21–39 years: ‘× increase’ = (3 + 1)/(0 + 1) = 4.00.

### Intra-species complexity of *P. falciparum*

#### Greater intra-species complexity of *P. falciparum* in larger pRBC volumes

**Fig. 3:**
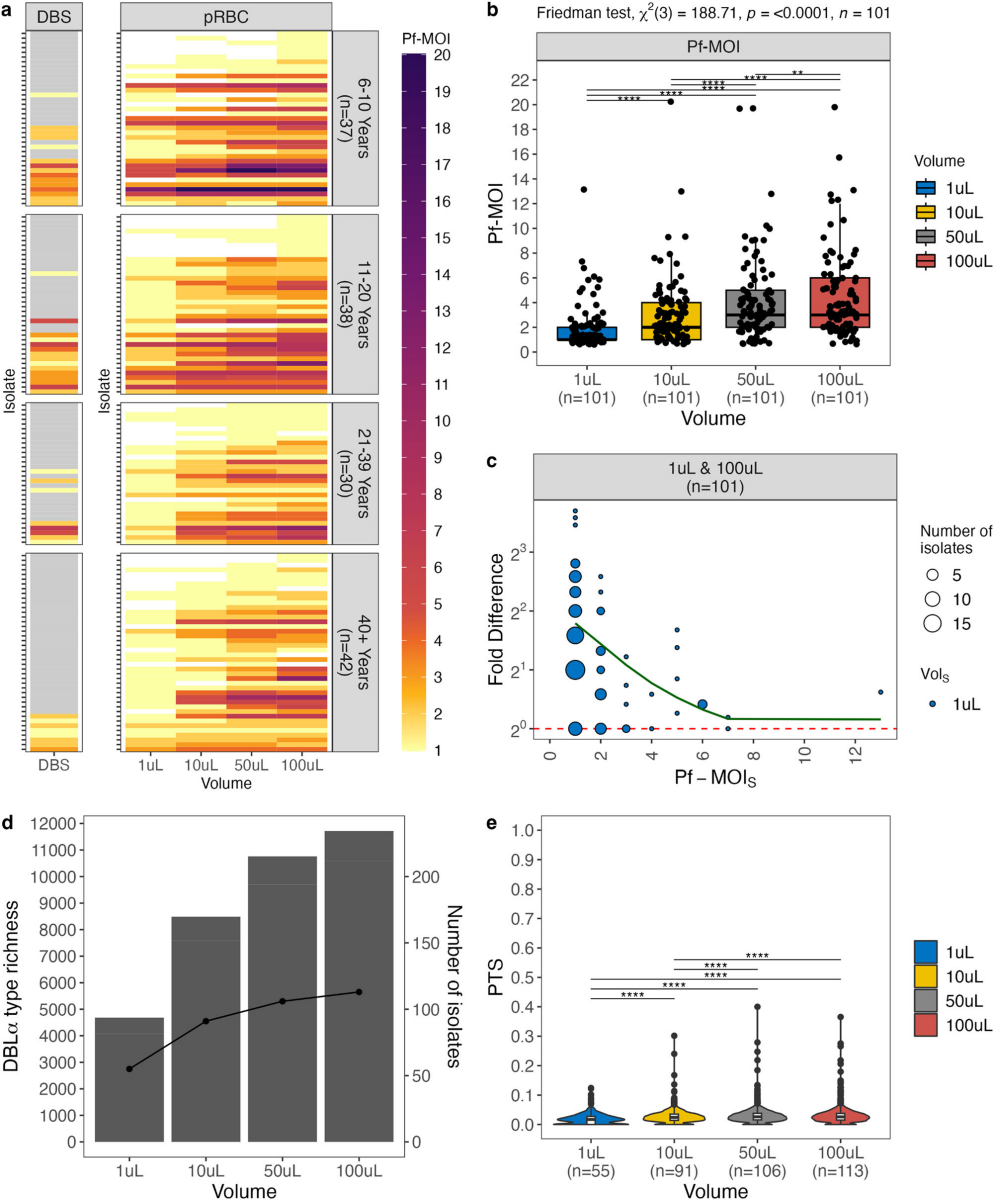
Higher levels of *P. falciparum* Pf-MOI were detected in larger pRBC volumes. **a** Shown here are only 147 isolates with Pf-MOI ≥ 1 in at least one pRBC volume, stratified by host age (6–10 years (*n* = 37 isolates), 11–20 years (*n* = 38 isolates), 21–39 years (*n* = 30 isolates), and ≥40 years (*n* = 42 isolates)). Isolates are ordered according to the order in Fig. 2. For DBS, isolates in grey were not *var*coded as these were negative by microscopy. **b** Boxplots are shown for isolates with Pf-MOI ≥ 1 for all four pRBC volumes (*n* = 101 isolates). Significance of pairwise pRBC comparisons was inferred with pairwise Wilcoxon signed rank tests, with levels of significance adjusted using the “holm” correction. **c** Non-linear relationship between Pf-MOI fold differences (FD_*Pf-MOI*_ in log scale) and the Pf-MOI measured from 1 μL-pRBC volumes (Pf-MOI_*S*_), shown for isolates with Pf-MOI ≥ 1 for all four pRBC volumes (*n* = 101 isolates). Red dashed line indicates no difference in Pf-MOI (FD_*Pf-MOI*_ = 1) while green solid line shows the smooth curve fitted with a generalised additive model. **d** Population-level metrics were estimated for isolates with Pf-MOI ≥ 1 with isolate repertoire size ≥ 20. Differences in DBLα type richness and number of infected individuals are shown, represented by bars and points, respectively. **e** Differences in genetic similarity between isolate repertoires by pairwise-type sharing (PTS), where most distributions were significantly different, with the exception of 50 μL *vs* 100 μL. Values are available in Supplementary Table 9. Statistical analysis was conducted with the Kruskal-Wallis analysis of variance, followed by Dunn’s test for multiple comparisons. In box and violin plots, the central line in each box represents the median, the box represents the interquartile range (IQR) from the 25^th^ to 75^th^ centiles, the whiskers indicate the most extreme data points within 1.5x IQR, and dots display data points. In all plots, significance symbols *, **, ***, and **** represent *p*-value significance levels of 0.05, 0.01, 0.001, and 0.0001, respectively.

Many isolates had greater *P. falciparum* strain complexity with larger blood sampling (Fig. 3a, Supplementary. Tables 5-6). Using a fingerprinting method (*var*coding^22^) shown to more accurately estimate *P. falciparum* MOI (Pf-MOI) in high-transmission areas^46^, 101 isolates had Pf-MOI ≥ 1 across all four pRBC volumes. Distributions of Pf-MOI were significantly different (Friedman test*, p-value* < *0.0001*, *n* = *101*), with the largest increase observed between 1μL- and 100μL-pRBC with median Pf-MOI of 1 and 3, respectively (pairwise Wilcoxon signed rank test*, p-value* < *0.0001*, *n* = *101*) (Fig. 3b). Concordance was high for Pf-MOI from 50 μL and 100 μL, suggesting near-saturation with these larger volumes (Supplementary Fig. 2) though Pf-MOI distributions remained significantly different (pairwise Wilcoxon signed rank test, *p-value* < *0.01*, *n* = *101*) (Fig. 3b). Most DBLα types found in 1 μL-pRBC were also present in the larger volumes (Supplementary Figs. 3–5). High concordance in Pf-MOI was estimated between 40 repeat isolates at all volumes and Pf-MOI distributions between repeats were not significantly different, supporting a robust approach in this study (Supplementary Fig. 6).

#### Non-linear relationships between Pf-MOI fold differences and initial Pf-MOI

For isolates with Pf-MOI = 0 in a pRBC volume but Pf-MOI ≥ 1 in a different volume (i.e. Pf-MOI_S_ = 0 and Pf-MOI_L_ > 0 OR Pf-MOI_L_ = 0 and Pf-MOI_S_ > 0), these Pf-MOI values mostly differed by one, with a minority of isolates ( < 8%) differing by two or three (Supplementary Fig. 7, Supplementary Table. 7). Taking a comparison between 1 μL- and 100 μL-pRBC as an example, of the 42 isolates with Pf-MOI_1μL_ = 0, we observed 9.50% (4/42), 81.0% (34/42), 4.80% (2/42), and 4.80% (2/42) of isolates showed Pf-MOI_100μL_ of 0, 1, 2, and 3, respectively. For the 101 isolates with Pf-MOI ≥ 1 across all four pRBC volumes, relationships between fold differences in Pf-MOI (FD_*Pf-MOI*_) and the Pf-MOI in the smaller compared pRBC volumes (Pf-MOI_*S*_) were non-linear (Fig. 3c, Supplementary Fig. 8). Relatively small FD_*Pf-MOI*_ values were observed for MOI comparisons between 50 μL- *vs* 100 μL-pRBC, suggesting again that sampling was almost saturated at these volumes sufficient to capture the complexity of *P. falciparum* multiclonal infections in our study population. These non-linear correlations can be considered for use to computationally adjust Pf-MOI values estimated for DBS samples in large-scale field surveillance. A power analysis based on proportions of Pf-MOI > 1 isolates in each pair of 1 μL *vs* larger pRBC volume determined that there was sufficient data for comparison of different pRBC volumes when all isolates were considered, but larger sample sizes are needed for analyses stratified by host ages (Supplementary Table. 8).

#### Increased *P. falciparum* complexity added to richness while infections remained largely unrelated

Our observations can be used to evaluate potential differences in epidemiological and population genetics metrics when variable blood volumes are sampled for a same set of individuals. Increased DBLα type richness was observed with larger pRBC sampling volumes (Fig. 3d) while DBLα type sharing between isolate repertoires remained low for all four pRBC volumes, indicating minimal genetic overlap (Fig. 3e, Supplementary Table 9). Thus, not only did the larger sampling of blood volume detect greater complexity of strains within infections in the form of higher Pf-MOI, this additional complexity contributed newly-detected diversity in the population such that we did not find clonal or highly-related parasites between isolates, as evidenced from rising cumulative diversity (Supplementary Fig. 9).

#### Shallow but broad sampling *vs* deep but restricted sampling: is one approach enough for malaria surveillance?

We further investigated whether the antigenic diversity in a shallow but broad sampling of the larger population (DBS) can be detected in a deep but restricted sampling of a subset of individuals (pRBC). We define ‘shallow but broad sampling’ referring to sampling many individuals but with smaller blood volumes, as opposed to ‘deep but restricted sampling’ when referring to sampling MOI or complexity within a few individuals but with larger blood volumes (Fig. 1).

To explore this, we included DBS samples from 1809 individuals from our larger cross-sectional survey in Bongo collected during the same surveyed time point in November 2020. Of these, 263 were positive for asymptomatic *P. falciparum* infections by microscopy. When comparing DBLα types recovered from DBS of 263 individuals and 100 μL-pRBC from 113 individuals, only 6821 (32.1%) of the total 21,222 DBLα types was seen in combined DBS and 100 μL-pRBC samples (Supplementary Fig. 10, Supplementary Table 10). Deep vertical sampling of the 113 individuals using 100 μL-pRBC volumes recovered 4894 (23.1%) of the total DBLα types that were not recovered in broad sampling of DBS of the 263 individuals at relatively shallow depths. The remainder of 9507 (44.8%) of total DBLα types was recovered by DBS sampling but not seen in the pRBC sampling, indicating that diversity of DBLα types in the larger DBS population was not fully captured by the diversity detected in a few individuals. Finding subsets of DBLα types exclusively from different sampling strategies suggests that both broad and deep sampling are necessary and complementary to more accurately reflect true population-level metrices of diversity and MOI.

### Inter- and intra-species complexity of *Plasmodium* spp.

We report on infection complexity for three minor species *P. malariae* (Pm-MOI), *P. ovale curtisi* (Poc-MOI), and *P. ovale wallikeri* (Pow-MOI) in 100 μL-pRBC samples of individuals previously identified as parasite positive by *18S rRNA*. For 26 individuals infected with *P. malariae*, microsatellite data revealed a median Pm-MOI of 2 [range: 1–4]. Of the ten individuals infected with *P. ovale* spp., there were eight *P. ovale curtisi*, one *P. ovale wallikeri*, and one double-species infection based on the *P. ovale* spp. tryptophan-rich antigen gene (*potra*), with fragment sizes reporting Poc-MOI and/or Pow-MOI of 1.

**Fig. 4:**
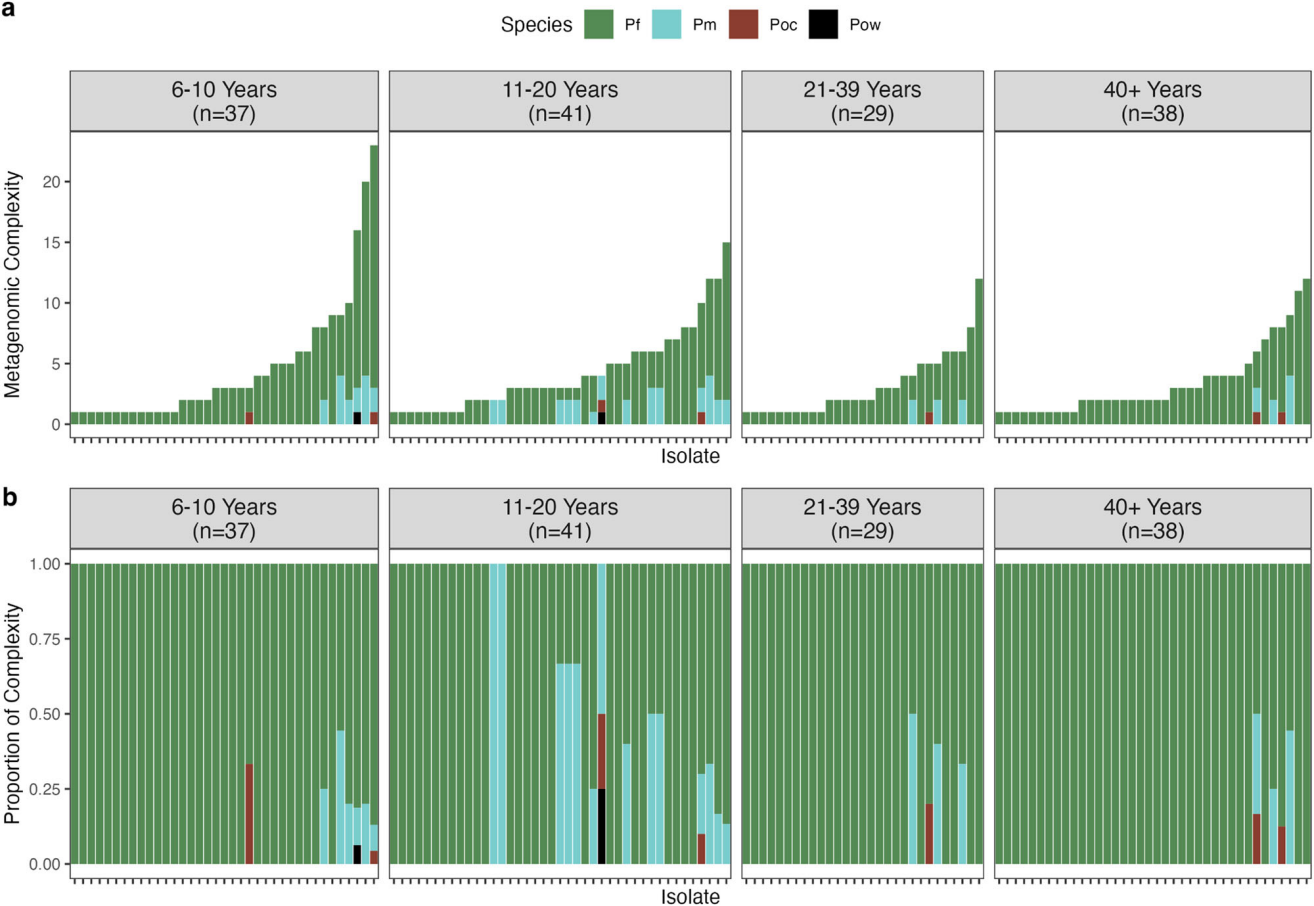
Malaria infection complexity comprising of single-, double-, and triple-species infections with varying MOI levels. Isolates are ordered in increasing metagenomic complexity, i.e., total MOI from four combined *Plasmodium* spp. **a** Metagenomic complexity calculated from the summation of estimated MOI across all four species of *P. falciparum* (Pf ), *P. malariae* (Pm), *P. ovale curtisi* (Poc), and *P. ovale wallikeri* (Pow). **b** Proportion of complexity representing the proportion of MOI contributed per species.

For 146 isolates with any MOI data, the summation of estimated MOI across all four species is expressed as metagenomic complexity (Fig. 4a), showing single-, double-, or triple-species infections (Fig. 4b). Whilst the one triple Pm-Poc-Pow infection reflected an absence of *P. falciparum* (i.e., *var*coding data, Pf-MOI = 0), *P. falciparum* was detected in this isolate with *18S rRNA* PCR, confirming a quadruple-species infection. The absence of *P. falciparum* in the two Pm-only infections was supported by *18S rRNA* PCR (i.e., not detected) and *var*coding data (i.e., Pf-MOI = 0).

Extreme metagenomic complexity with summed MOI from 10 to 23 was seen in a subset of individuals in every age group (Fig. 4a), occurring at a prevalence of 5.85% (11/188) in this survey of 188 individuals. Most of these were in the groups of children (4 individuals) and adolescents (4 individuals). These individuals had as many as 7 to 20 different *P. falciparum* antigenic strains, 2 to 4 *P. malariae* genotypes by microsatellites, and/or 1 each of *P. ovale curtisi*/*P. ovale walikeri* antigenic strains by *potra*. It is unclear why extreme complexity was found in these individuals, comprising nine males and two females. Inspection of association between metagenomic complexity and human host or spatial characteristics did not yield any notable findings (Supplementary Table 11).

### Impact on malaria surveillance and elimination

By comparing DBS or 1 uL-pRBC to larger sampling of 100uL-pRBC, this study quantified the underestimation of prevalence and metagenomic complexity in current protocols employing small blood volumes. We describe below a case example of how we can potentially use these quantified factors to adjust estimates from the larger survey of DBS samples of 1809 individuals in Bongo, of which 1455 were aged ≥6 years.

**Fig. 5:**
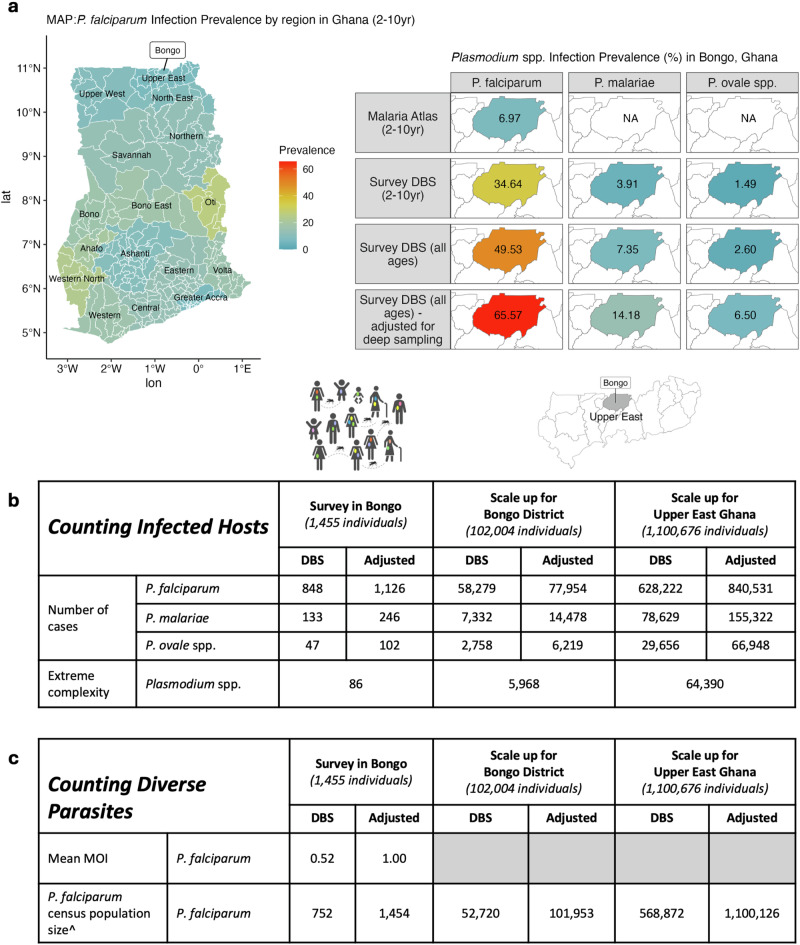
A case example of the impact of underestimated complexity on the large reservoir in the Bongo District and the Upper East Region of Northern Ghana. In this figure, ‘Adjusted’ represents adjusted estimates to account for larger volume sampling (i.e., 100 μL). **a** Malaria Atlas Project (MAP) report of *P. falciparum* infection prevalence by region in Ghana in children 2–10 years (in year 2020), and comparison of *P. falciparum* prevalences in Bongo in the year 2020 estimated by MAP for Bongo in Ghana in children 2–10 years (PfPR_2-10_ data for year 2020), by *18S rRNA* in our larger DBS survey in children 2–10 years, in our larger DBS survey in all host ages, and adjusted for larger volume sampling in all host ages. Also shown are prevalences for minor species *P. malariae* and *P. ovale* spp. that are not available in MAP. **b** Impact on metrics that count infected hosts. Prevalence and adjusted prevalence estimated from the larger DBS survey in individuals ≥6 years were scaled up to approximate the number of malaria cases in the Bongo DBS survey, Bongo District, and the Upper East Region of Ghana, detailed in Supplementary Table 12. Similarly, the numbers of heavily-infected individuals with extreme metagenomic complexity in these regions were approximated with the 5.85% prevalence reported in this study. **c** Impact on metrics that count diverse parasites. Similarly, mean Pf-MOI and adjusted mean Pf-MOI, estimated from the larger DBS survey in infected individuals ≥6 years, were scaled up to approximate the *P. falciparum* census population size in the Bongo DBS survey, Bongo District, and the Upper East Region of Ghana without host age stratification. Tables are available in accessible format in Supplementary Tables 13–15.

Estimated through geostatistical modelling methods applied to rapid diagnostic tests or microscopy data, the Malaria Atlas Project (MAP)^56,57^ reports a standardised *P. falciparum* prevalence of 6.97% in Bongo in the Upper East Region of Ghana, based on the proportion of children aged 2–10 years in the year 2020. Employing molecular methods on DBS samples collected in the same year 2020, we estimated higher *P. falciparum* crude prevalence in the same age group of children aged 2–10 years (186/537, 34.64%) and even higher crude prevalence when individuals of all ages were inspected (896/1809, 49.53%) (Fig. 5a). Following adjustments to account for larger volume sampling when individuals of all ages were inspected, a 65.57% *P. falciparum* crude prevalence was estimated for Bongo. This analysis compares data from the same sampling year (2020), but we could not account for potential differences in sampling seasons (e.g., malaria peak season *vs*. MAP’s yearly average) due to the lack of availability of monthly prevalence data from MAP, which may lead to a smaller underestimation than described. This study has additionally made available crude prevalence data for minor *Plasmodium* spp. currently unavailable in MAP.

To illustrate the relevance of these findings to malaria surveillance and elimination, we scaled up prevalence and *P. falciparum* complexity data to the district and regional levels in Bongo and the Upper East Region of Ghana, respectively, based on a 2021 Population and Housing census by the Ghana Statistical Service (GSS)^66,67^. Scale up of difference in prevalence of *P. falciparum*, *P. malariae*, and *P. ovale* spp. (Fig. 5b) predicted that tens of thousands of *Plasmodium* spp. infections would be missed in Bongo District from using DBS, and hundreds of thousands would be missed in the wider Upper East Region of Ghana (Supplementary Table 12). In a population, thousands of these individuals are expected to be heavily infected with highly-complex infections, representing a substantial reservoir. This study provides a sampling frame to look for these individuals for further exploration.

Whilst parasite prevalence and clinical incidence count the number of infected human hosts (Fig. 5a, b), other metrics such as MOI and parasite census population size count the number of diverse parasite genomes in infections (Fig. 5c). A mean Pf-MOI of 3.04 was calculated from *var*coding of DBS samples from 247 individuals microscopy-positive for *P. falciparum*. Subsequently, mean Pf-MOI was adjusted to 5.89 to account for larger volume sampling, resulting in larger *P. falciparum* census population sizes in Bongo District and the Upper East Region of Ghana.

We make clear that actual design and implementation of such a strategy will require careful considerations taking into account sampling season and consolidating areas with similar malaria epidemiologies, which was not undertaken in this study. Such strategies would also require us to treat groups of individuals receiving targeted malaria control interventions (e.g., SMC) separately in analysis.

## Discussion

Our metagenomic approach of sampling variable blood volumes for *Plasmodium* spp. in Bongo, Ghana highlighted the complexity of infections in this area in West Africa. This endemic area is in one of the three ecological zones in Ghana (i.e. ‘Sahel Savannah zone’)^68^ and has been described as ‘Northern Sahelian Ghana’^31^. Application of our proposed metagenomic sampling strategy may be broadly considered in similar ecologies in West Africa with high seasonal malaria transmission^69,70^. This contemporary deep dive into *Plasmodium* spp. infection complexity, post-multiple interventions^22^ found *P. falciparum* as the dominant species with minor species *P. malariae*, *P. ovale curtisi*, and *P. ovale wallikeri* present, generally at submicroscopic levels. *P. vivax* was undetected in the Bongo population, likely due to the protection of Duffy-negativity against sustained endemic *P. vivax* infections in West African human hosts^71^. Scaling up to district and regional level, we predicted that most individuals of all ages are infected with *Plasmodium* spp. and current DBS surveys are substantially underestimating the prevalence of minor species infections.

Our report of single-, double-, and triple-species infections comprising varying MOI levels per species shows that highly complex infections are common in this asymptomatic reservoir in Ghana. Regarding the diversity of minor *Plasmodium* spp., our reliance on a limited set of markers of varying sensitivities (microsatellites for *P. malariae* and *potra* for *P. ovale* spp.) may substantially underestimate their true diversity, especially in contrast to our use of highly diverse variant antigen genes (*var* genes) for *P. falciparum*. Of note, we observed a subset of individuals with extreme metagenomic complexity of infection in all age classes. Whether susceptibility to extreme mixed infections is due to behavioural, host genetic, immunological, and/or environmental factors warrants further investigation. This observation is reminiscent of ‘wormy’ people observed in helminth ecology/epidemiology studies^72^ and susceptibility may be influenced by anopheline preferential biting behaviour^39^. These heavily-infected individuals warrant further investigation and may be examined longitudinally and in terms of host genomics and immunological profiles to inform on infection history.

Given the elevated diversity and frequent multiclonal infections in the malaria reservoir in high-transmission areas, metrics of parasite prevalence and clinical incidence that use the infected human host as the unit of measurement do not capture the complexity of infections in the asymptomatic reservoir. Instead, our study provides the evidence to develop protocols to obtain better estimates of MOI and parasite census population size^22^ as endpoints for evaluating interventions. This study design may serve as a prototype to design future surveillance of other high-transmission areas that may need to be run concurrently with larger malaria surveys to facilitate MOI correction in a cost-effective approach. Further, following up on this idea of viewing infections as metagenomic entities, there is also avenue for future work to examine the possibility of obtaining relative abundance values to explore the calculation of other metagenomic diversity metrics (e.g., evenness, dominance, dissimilarity), potentially from read support per amplified marker and normalised across the different markers and approaches.

We have illustrated the impact of scaling prevalence of infected hosts based on larger blood volume sampling in Bongo District and the larger Upper East Region of Ghana. Scaling up on within-host diversity must consider the observed non-linear relationship between fold differences in Pf-MOI and the Pf-MOI in the smaller compared pRBC volumes, with the obvious implication that DBS estimates cannot be corrected by a single factor of difference. Instead, such a factor must be calculated using a measured distribution, where adjustments are mostly needed for isolates with Pf-MOI in the 1 to 3 range. Larger sample sizes stratified by age, gender, and relevant heterogeneities to an endemic area will be necessary to explore these non-linear relationships further as will Bayesian approaches^56^. This type of scale-up of a study to multiple spatial sites counting both infected hosts and genetically-diverse parasites per host has broad applications for geographic information system (GIS) mapping, such as for the Malaria Atlas Project^56^. Our findings make the case that both broad and deep sampling of all ages in sentinel sites is needed to achieve accurate representation of an asymptomatic reservoir.

Malaria elimination in Africa requires a focus on the reservoir, characterised by low-density subclinical infections. Yet, there have been limited investigations of these low-density subclinical infections of *Plasmodium* spp. in general^73–76^, with the majority of current molecular methods developed for high-density, clinical samples. In this context, our study serves as a warning of the extent of the malaria problem in terms of prevalence and diversity in the reservoir of infection in all ages in high-burden, high-transmission areas in Africa. Data presented also point to the need for a change in perspective and approaches in malaria genomic surveillance, so that we view malaria infections holistically as the complex infections that they are rather than the examination of each species individually^19,77–80^. In doing so, we can better study the impact of transmission-reducing interventions such as indoor residual spraying (IRS) and vaccines on the reservoir^22^. Of further interest would be to also consider how other factors such as age of samples post-collection or sample storage conditions^81^ can affect PCR sensitivity and estimation of metagenomic complexity.

Additionally, metagenomics offers a way to study emerging drug resistance in rare strains and minor species conferred through current treatment regimens optimised for *P. falciparum*^82–84^. With prolonged SMC and widespread use of antimalarials to *P. falciparum* in communities, the probability of drug resistance is likely to increase silently in *P. malariae* and *P. ovale* spp., pointing to the need for deep sampling of individuals in the reservoir. Such mutations have already been found with investigators using whole genome amplification and large volumes of blood from clinical cases where parasitaemias are significantly higher^85^. Metagenomics further allows us a means to examine complex interactions among species (e.g., ref. ^5^) revealed by epidemiological patterns as well as emergence of minor species with relapse capability. To achieve this, accurate measurement of infection complexity is crucial by taking on board metagenomic approaches to sampling. Broadly, our results are also relevant to those doing surveillance of Haemosporidian parasites of other hosts infected with multiple species and genotypes.

## Supplementary information

Supplementary Information

Reporting Summary

## Data Availability

*P. falciparum* DBLα tag sequences have been deposited to Genbank under the accession PRJNA1266761. This Targeted Locus Study project has been deposited at DDBJ/EMBL/GenBank under the accession KIYA00000000. The version described in this paper is the first version, KIYA01000000. Protocols and primer sequences are available on GitHub (https://github.com/UniMelb-Day-Lab/SpeciesSpecific_18S_rRNA_PCR; https://github.com/UniMelb-Day-Lab/Pfalciparum_varDBLalpha_PCR; https://github.com/UniMelb-Day-Lab/Pmalariae_Microsat_PCR; https://github.com/UniMelb-Day-Lab/Povale_potra_PCR). DBLα type sequences and data tables underlying results are available on GitHub (https://github.com/mh-tan/Metagenomic_Complexity_Plasmodium). The individual age data are not publicly available due to ethical reasons. Requests for data on individual age classes corresponding to *P. falciparum* isolates should be made by contacting the Malaria Reservoir Study Team represented by Prof. Karen Day (karen.day@unimelb.edu.au; Response timeframe: ~1 month), in order to discuss how these data will be utilised for academic or research purposes and, if appropriate, to identify opportunities for collaboration.

## Code availability

A collection of custom R scripts used to generate results are available on GitHub at: https://github.com/mh-tan/Metagenomic_Complexity_Plasmodium, digital resource identifier on Zenodo^86^: 10.5281/zenodo.15750983.

## References

[1] Galen, S. C. et al. The polyphyly of *Plasmodium*: comprehensive phylogenetic analyses of the malaria parasites (order Haemosporida) reveal widespread taxonomic conflict. *R. Soc. Open Sci.* **5**, 171780 (2018). 29892372 10.1098/rsos.171780 PMC5990803

[2] Sharp, P. M., Plenderleith, L. J. & Hahn, B. H. Ape Origins of Human Malaria. *Annu Rev. Microbiol* **74**, 39–63 (2020). 32905751 10.1146/annurev-micro-020518-115628 PMC7643433

[3] Fuehrer, H.-P., Campino, S. & Sutherland, C. J. The primate malaria parasites *Plasmodium malariae*, *Plasmodium brasilianum* and *Plasmodium ovale* spp.: genomic insights into distribution, dispersal and host transitions. *Malar. J.* **21**, 138 (2022). 35505317 10.1186/s12936-022-04151-4 PMC9066925

[4] Guimarães, L. O. et al. The genetic diversity of *Plasmodium malariae* and *Plasmodium brasilianum* from human, simian and mosquito hosts in Brazil. *Acta Trop.* **124**, 27–32 (2012). 22705349 10.1016/j.actatropica.2012.05.016

[5] Bruce, M. C. et al. Cross-species interactions between malaria parasites in humans. *Science ((1979))* **287**, 845–848 (2000). 10.1126/science.287.5454.845 10657296

[6] Bruce, M. C., Macheso, A., McConnachie, A. & Molyneux, M. E. Comparative population structure of *Plasmodium malariae* and *Plasmodium falciparum* under different transmission settings in Malawi. *Malar. J.* **10**, 38 (2011). 21314950 10.1186/1475-2875-10-38 PMC3050775

[7] Yman, V. et al. Persistent transmission of *Plasmodium malariae* and *Plasmodium ovale* species in an area of declining *Plasmodium falciparum* transmission in eastern Tanzania. *PLoS Negl. Trop. Dis.* **13**, e0007414 (2019). 31136585 10.1371/journal.pntd.0007414 PMC6555537

[8] Gumbo, A. et al. Occurrence and Distribution of Nonfalciparum Malaria Parasite Species Among Adolescents and Adults in Malawi. *J. Infect. Dis.* **225**, 257–268 (2022). 34244739 10.1093/infdis/jiab353 PMC8763954

[9] Mayxay, M., Pukrittayakamee, S., Newton, P. N. & White, N. J. Mixed-species malaria infections in humans. *Trends Parasitol.* **20**, 233–240 (2004). 15105024 10.1016/j.pt.2004.03.006

[10] WHO. World Malaria Report 2024. (World Health Organization, 2024).

[11] Lindblade, K. A., Steinhardt, L., Samuels, A., Kachur, S. P. & Slutsker, L. The silent threat: asymptomatic parasitemia and malaria transmission. *Expert Rev. Anti Infect. Ther.* **11**, 623–639 (2013). 23750733 10.1586/eri.13.45

[12] Pinkevych, M. et al. The Dynamics of Naturally Acquired Immunity to *Plasmodium falciparum* Infection. *PLoS Comput Biol.* **8**, e1002729 (2012). 23093922 10.1371/journal.pcbi.1002729 PMC3475668

[13] Tran, T. M. et al. An Intensive Longitudinal Cohort Study of Malian Children and Adults Reveals No Evidence of Acquired Immunity to *Plasmodium falciparum* Infection. *Clin. Infect. Dis.* **57**, 40–47 (2013). 23487390 10.1093/cid/cit174 PMC3669526

[14] Owusu-Agyei, S., et al. Incidence of symptomatic and asymptomatic *Plasmodium falciparum* infection following curative therapy in adult residents of northern Ghana. *Am. J. Trop. Med. Hyg.* **65**, 197–203 (2001). 11561704 10.4269/ajtmh.2001.65.197

[15] Lai, M. Y. et al. High incidence of *Plasmodium knowlesi* malaria compared to other human malaria species in several hospitals in Malaysia. **38**, 248–253 (2021). 10.47665/tb.38.3.065 34362867

[16] Singh, B., et al. A large focus of naturally acquired *Plasmodium knowlesi* infections in human beings. *Lancet* **363**, 1017–1024 (2004). 15051281 10.1016/S0140-6736(04)15836-4

[17] Culleton, R., Pain, A. & Snounou, G. *Plasmodium malariae*: the persisting mysteries of a persistent parasite. *Trends Parasitol.* **39**, 113–125 (2023). 36517330 10.1016/j.pt.2022.11.008

[18] Rutledge, G. G. et al. *Plasmodium malariae* and *P. ovale* genomes provide insights into malaria parasite evolution. *Nature* **542**, 101–104 (2017). 28117441 10.1038/nature21038 PMC5326575

[19] Carey-Ewend, K. et al. Population genomics of *Plasmodium ovale* species in sub-Saharan Africa. *Nat. Commun.* **15**, 10297 (2024). 39604397 10.1038/s41467-024-54667-3 PMC11603351

[20] Sutherland, C. J. et al. Two Nonrecombining Sympatric Forms of the Human Malaria Parasite *Plasmodium ovale* Occur Globally. *J. Infect. Dis.* **201**, 1544–1550 (2010). 20380562 10.1086/652240

[21] Snounou, G., Sharp, P. M. & Culleton, R. The two parasite species formerly known as *Plasmodium ovale*. *Trends Parasitol.* **40**, 21–27 (2024). 38040603 10.1016/j.pt.2023.11.004

[22] Tiedje, K. E. et al. Measuring changes in *Plasmodium falciparum* census population size in response to sequential malaria control interventions. *Elife* **12**, RP91411 (2023). 10.7554/eLife.91411 PMC12563550 41150052

[23] Andolina, C. et al. Sources of persistent malaria transmission in a setting with effective malaria control in eastern Uganda: a longitudinal, observational cohort study. *Lancet Infect. Dis.* **21**, 1568–1578 (2021). 34146476 10.1016/S1473-3099(21)00072-4 PMC8554388

[24] Zhang, X. & Deitsch, K. W. The mystery of persistent, asymptomatic *Plasmodium falciparum* infections. *Curr. Opin. Microbiol* **70**, 102231 (2022). 36327690 10.1016/j.mib.2022.102231 PMC10500611

[25] Zhan, Q., He, Q., Tiedje, K. E., Day, K. P. & Pascual, M. Hyper-diverse antigenic variation and resilience to transmission-reducing intervention in falciparum malaria. *Nat. Commun.* **15**, 7343 (2024). 39187488 10.1038/s41467-024-51468-6 PMC11347654

[26] Smith, T. et al. Age dependence of the multiplicity of *Plasmodium falciparum* infections and of other malariological indices in an area of high endemicity. *Trans. R. Soc. Trop. Med Hyg.* **93**, 15–20 (1999). 10450421 10.1016/s0035-9203(99)90322-x

[27] Ruybal-Pesántez, S. et al. Age-specific patterns of DBLα *var* diversity can explain why residents of high malaria transmission areas remain susceptible to *Plasmodium falciparum* blood stage infection throughout life. *Int J. Parasitol.* **52**, 721–731 (2022). 35093396 10.1016/j.ijpara.2021.12.001 PMC9339046

[28] Cena, J. A. de, Zhang, J., Deng, D., Damé-Teixeira, N. & Do, T. Low-Abundant Microorganisms: The Human Microbiome’s Dark Matter, a Scoping Review. *Front. Cell Infect. Microbiol.* **11**, 10.3389/fcimb.2021.689197 (2021). 10.3389/fcimb.2021.689197 PMC8201079 34136418

[29] Mächler, E., Deiner, K., Spahn, F. & Altermatt, F. Fishing in the Water: Effect of Sampled Water Volume on Environmental DNA-Based Detection of Macroinvertebrates. *Environ. Sci. Technol.* **50**, 305–312 (2016). 26560432 10.1021/acs.est.5b04188

[30] Staley, C. et al. Evaluation of water sampling methodologies for amplicon-based characterization of bacterial community structure. *J. Microbiol Methods* **114**, 43–50 (2015). 25956022 10.1016/j.mimet.2015.05.003

[31] Ansah, P. O. et al. Evaluation of pilot implementation of seasonal malaria chemoprevention on morbidity in young children in Northern Sahelian Ghana. *Malar. J.* **20**, 440 (2021). 34794431 10.1186/s12936-021-03974-x PMC8600740

[32] Tiedje, K. E. et al. Seasonal Variation in the Epidemiology of Asymptomatic *Plasmodium falciparum* Infections across Two Catchment Areas in Bongo District, Ghana. * Am. Soc. Tropical Med. Hyg.* **97**, 199–212 (2017). 10.4269/ajtmh.16-0959 PMC5508908 28719306

[33] Tiedje, K. E. et al. Indoor residual spraying with a non-pyrethroid insecticide reduces the reservoir of *Plasmodium falciparum* in a high-transmission area in northern Ghana. *PLOS Glob. Public Health* **2**, e0000285–e0000285 (2022). 35600674 10.1371/journal.pgph.0000285 PMC9121889

[34] WHO. *Preparation of Blood Spots on Filter Paper*. (2016).

[35] Snounou, G. et al. High sensitivity of detection of human malaria parasites by the use of nested polymerase chain reaction. *Mol. Biochem Parasitol.* **61**, 315–320 (1993). 8264734 10.1016/0166-6851(93)90077-b

[36] Bruce, M. C., Macheso, A., Galinksi, M. R. & Barnwell, J. W. Characterization and application of multiple genetic markers for *Plasmodium malariae*. *Parasitology* **134**, 637–650 (2006). 17140466 10.1017/S0031182006001958 PMC1868962

[37] Mathema, V. B. et al. Polymorphic markers for identification of parasite population in *Plasmodium malariae*. *Malar. J.* **19**, 48 (2020). 31992308 10.1186/s12936-020-3122-2 PMC6988369

[38] Anderson, T. J. C., Su, X.-Z., Bockarie, M., Lagog, M. & Day, K. P. Twelve microsatellite markers for characterization of *Plasmodium falciparum* from finger-prick blood samples. *Parasitology* **119**, 113–125 (1999). 10466118 10.1017/s0031182099004552

[39] Matschiner, M. & Salzburger, W. TANDEM: integrating automated allele binning into genetics and genomics workflows. *Bioinformatics* **25**, 1982–1983 (2009). 19420055 10.1093/bioinformatics/btp303

[40] Šlapeta, J., Sutherland, C. J. & Fuehrer, H.-P. Calling them names: variants of *Plasmodium ovale*. *Trends Parasitol.* **40**, 205–206 (2024). 38160179 10.1016/j.pt.2023.12.010

[41] Markus, M. B. Type material of *Plasmodium ovale* sensu lato. *S Afr. J. Infect. Dis.* **39**, 615 (2025). 10.4102/sajid.v39i1.615 PMC11019055 38628423

[42] Snounou, G., Sharp, P. M. & Culleton, R. Appropriate naming of the two *Plasmodium ovale* species. *Trends Parasitol.* **40**, 207–208 (2024). 38272740 10.1016/j.pt.2024.01.004

[43] Oguike, M. C. et al. *Plasmodium ovale curtisi* and *Plasmodium ovale wallikeri* circulate simultaneously in African communities. *Int J. Parasitol.* **41**, 677–683 (2011). 21315074 10.1016/j.ijpara.2011.01.004 PMC3084460

[44] Tanomsing, N. et al. Genetic Marker Suitable for Identification and Genotyping of *Plasmodium ovale curtisi* and *Plasmodium ovale wallikeri*. *J. Clin. Microbiol* **51**, 4213–4216 (2013). 24068009 10.1128/JCM.01527-13 PMC3838052

[45] Ghansah, A. et al. Comparison of molecular surveillance methods to assess changes in the population genetics of *Plasmodium falciparum* in high transmission. *Front. Parasitol.* **v2**, 1067966. 10.3389/fpara.2023.1067966 (2023). 10.3389/fpara.2023.1067966 PMC10686283 38031549

[46] Labbé, F. et al. Neutral vs. non-neutral genetic footprints of *Plasmodium falciparum* multiclonal infections. *PLoS Comput Biol.* **19**, e1010816 (2023). 36595546 10.1371/journal.pcbi.1010816 PMC9838855

[47] Bull, P. C. et al. *Plasmodium falciparum* Variant Surface Antigen Expression Patterns during Malaria. *PLoS Pathog.* **1**, e26 (2005). 16304608 10.1371/journal.ppat.0010026 PMC1287908

[48] Taylor, H. M., Kyes, S. A., Harris, D., Kriek, N. & Newbold, C. I. A study of *var* gene transcription in vitro using universal *var* gene primers. *Mol. Biochem Parasitol.* **105**, 13–23 (2000). 10613695 10.1016/s0166-6851(99)00159-0

[49] He, Q. et al. Networks of genetic similarity reveal non-neutral processes shape strain structure in *Plasmodium falciparum*. *Nat. Commun.* **9**, 1817 (2018). 29739937 10.1038/s41467-018-04219-3 PMC5940794

[50] Ruybal-Pesántez, S. et al. Population genomics of virulence genes of *Plasmodium falciparum* in clinical isolates from Uganda. *Sci. Rep.* **7**, 11810 (2017). 28924231 10.1038/s41598-017-11814-9 PMC5603532

[51] Pilosof, S. et al. Competition for hosts modulates vast antigenic diversity to generate persistent strain structure in *Plasmodium falciparum*. *PLoS Biol.* **17**, e3000336–e3000336 (2019). 31233490 10.1371/journal.pbio.3000336 PMC6611651

[52] Barry, A. E. et al. Population Genomics of the Immune Evasion (*var*) Genes of *Plasmodium falciparum*. *PLoS Pathog.* **3**, e34–e34 (2007). 17367208 10.1371/journal.ppat.0030034 PMC1828697

[53] Rask, T. S., Hansen, D. A., Theander, T. G., Gorm Pedersen, A. & Lavstsen, T. *Plasmodium falciparum* Erythrocyte Membrane Protein 1 Diversity in Seven Genomes – Divide and Conquer. *PLoS Comput Biol.* **6**, e1000933 (2010). 20862303 10.1371/journal.pcbi.1000933 PMC2940729

[54] Wood, S. N. Fast Stable Restricted Maximum Likelihood and Marginal Likelihood Estimation of Semiparametric Generalized Linear Models. *J. R. Stat. Soc. Ser. B Stat. Methodol.* **73**, 3–36 (2011).

[55] Global ADMinistrative Areas (GADM). https://gadm.org/download_country.html.

[56] Weiss, D. J. et al. Mapping the global prevalence, incidence, and mortality of *Plasmodium falciparum*, 2000-17: a spatial and temporal modelling study. * Lancet* **394**, 322–331 (2019). 31229234 10.1016/S0140-6736(19)31097-9 PMC6675740

[57] Malaria Atlas Project. https://malariaatlas.org/.

[58] R Core Team, R. R: A language and environment for statistical computing. (2013).

[59] Kassambara, A. rstatix: Pipe-Friendly Framework for Basic Statistical Tests. (2023).

[60] Signorell, A. DescTools: Tools for Descriptive Statistics. (2024).

[61] Champely, S. et al. pwr: Basic functions for power analysis. (2017).

[62] Babiker, H. A. et al. Random mating in a natural population of the malaria parasite *Plasmodium falciparum*. *Parasitology* **109**, 413–421 (1994). 7800409 10.1017/s0031182000080665

[63] Paul, R. E. L. et al. Mating Patterns in Malaria Parasite Populations of Papua New Guinea. *Science ((1979))* **269**, 1709–1711 (1995). 10.1126/science.7569897 7569897

[64] WHO. *A Framework for Malaria Elimination*. (World Health Organization, 2017).

[65] WHO. *Global Technical Strategy for Malaria 2016-2030*. (World Health Organization, 2015).

[66] Ghana Statistical Service, Population and Housing Census 2021 (PHC 2021), Version 1.0 of the public use dataset (December 2011), provided by the Ghana Statistical Service. www.statsghana.gov.gh.

[67] Thomas Brinkhoff: City Population. https://www.citypopulation.de/en/ghana/admin/.

[68] Ministry of Health (MOH) & Ghana Health Service (GHS). *National Malaria Elimination Strategic Plan (NMESP) of Ghana: 2024-2028*.

[69] Noland, G. S. et al. Malaria prevalence, anemia and baseline intervention coverage prior to mass net distributions in Abia and Plateau States, Nigeria. *BMC Infect. Dis.* **14**, 168 (2014). 24669881 10.1186/1471-2334-14-168 PMC3994282

[70] Bationo, C. et al. Malaria in Burkina Faso: A comprehensive analysis of spatiotemporal distribution of incidence and environmental drivers, and implications for control strategies. *PLoS One* **18**, e0290233- (2023). 37703223 10.1371/journal.pone.0290233 PMC10499254

[71] Twohig, K. A. et al. Growing evidence of *Plasmodium vivax* across malaria-endemic Africa. *PLoS Negl. Trop. Dis.* **13**, e0007140- (2019). 30703083 10.1371/journal.pntd.0007140 PMC6372205

[72] Schad, G. A. & Anderson, R. M. Predisposition to Hookworm Infection in Humans. *Science ((1979))* **228**, 1537–1540 (1985). 10.1126/science.4012307 4012307

[73] Canier, L. et al. Malaria PCR detection in Cambodian low-transmission settings: dried blood spots versus venous blood samples. *Am. J. Trop. Med Hyg.* **92**, 573 (2015). 25561570 10.4269/ajtmh.14-0614 PMC4350552

[74] Koepfli, C. Is qPCR always the most sensitive method for malaria diagnostic quality surveillance. *Malar. J.* **22**, 380 (2023). 38102649 10.1186/s12936-023-04822-w PMC10722660

[75] Mallika, I. et al. High-Throughput Ultrasensitive Molecular Techniques for Quantifying Low-Density Malaria Parasitemias. *J. Clin. Microbiol* **52**, 3303–3309 (2020). 10.1128/JCM.01057-14 PMC4313154 24989601

[76] Lau, Y.-L. et al. Specific, sensitive and rapid detection of human *Plasmodium knowlesi* infection by loop-mediated isothermal amplification (LAMP) in blood samples. *Malar. J.* **10**, 197 (2011). 21774805 10.1186/1475-2875-10-197 PMC3156799

[77] Brokhattingen, N. et al. Genomic malaria surveillance of antenatal care users detects reduced transmission following elimination interventions in Mozambique. *Nat. Commun.* **15**, 2402 (2024). 38493162 10.1038/s41467-024-46535-x PMC10944499

[78] Liu, Y. et al. Genetic diversity and population structure of *Plasmodium vivax* in Central China. *Malar. J.* **13**, 262 (2014). 25008859 10.1186/1475-2875-13-262 PMC4094906

[79] Auburn, S. et al. Genomic analysis of a pre-elimination Malaysian *Plasmodium vivax* population reveals selective pressures and changing transmission dynamics. *Nat. Commun.* **9**, 2585 (2018). 29968722 10.1038/s41467-018-04965-4 PMC6030216

[80] Fuehrer, H.-P. et al. *Plasmodium ovale* in Bangladesh: Genetic diversity and the first known evidence of the sympatric distribution of *Plasmodium ovale curtisi* and *Plasmodium ovale wallikeri* in southern Asia. *Int J. Parasitol.* **42**, 693–699 (2012). 22633951 10.1016/j.ijpara.2012.04.015

[81] Hwang, J. et al. Long-term storage limits PCR-based analyses of malaria parasites in archival dried blood spots. *Malar. J.* **11**, 339 (2012). 23043522 10.1186/1475-2875-11-339 PMC3507721

[82] Pimpat, Y. et al. Genetic analysis of the orthologous crt and mdr1 genes in *Plasmodium malariae* from Thailand and Myanmar. *Malar. J.* **19**, 315 (2020). 32867773 10.1186/s12936-020-03391-6 PMC7461347

[83] Siswantoro, H. et al. In Vivo and In Vitro Efficacy of Chloroquine against *Plasmodium malariae* and *P. ovale* in Papua, Indonesia. *Antimicrob. Agents Chemother.* **55**, 197–202 (2011). 20937779 10.1128/AAC.01122-10 PMC3019630

[84] Oriero, E. C. et al. *Plasmodium malariae* structure and genetic diversity in sub-Saharan Africa determined from microsatellite variants and linked SNPs in orthologues of antimalarial resistance genes. *Sci. Rep.* **12**, 21881 (2022). 36536036 10.1038/s41598-022-26625-w PMC9761029

[85] Joste, V. et al. *Plasmodium ovale* spp *dhfr* mutations associated with reduced susceptibility to pyrimethamine in sub-Saharan Africa: a retrospective genetic epidemiology and functional study. *Lancet Microbe* **5**, 669–678 (2024). 38761813 10.1016/S2666-5247(24)00054-5

[86] Tan, M. H. et al. Metagenomic complexity of high, seasonal transmission of *Plasmodium* spp. in asymptomatic carriers in Northern Sahelian Ghana [Data set]. Zenodo. 10.5281/zenodo.15750983 (2025). 10.1038/s43856-025-01088-y PMC12423318 40931024

