# Supplementary material for: Metagenomic complexity of high, seasonal transmission of Plasmodium spp. in asymptomatic carriers in Northern Sahelian Ghana
Source: Commun Med (Lond). 2025 Sep 10;5:386. doi: 10.1038/s43856-025-01088-y (PMC12423318; doi:10.1038/s43856-025-01088-y)
Supplement: Supplementary file 1 — Supplementary Information [file 43856_2025_1088_MOESM1_ESM.pdf]

Supplementary Information

Table of Contents

Supplementary Method 1. Estimation of whole blood (WB) and packed red blood cell (pRBC) volume equivalent in dried blood spots (DBS) 3

Supplementary Method 2. Adjusting prevalence, Pf-MOI, and *P. falciparum* census population size with scale up to larger regions in Bongo and Upper East Ghana. 5

Supplementary Figure 1. Microscopic *P. falciparum* densities were significantly lower in asymptomatic isolates compared to clinical isolates during the same epidemiological survey. .. 6

Supplementary Table 1. Dataset sizes following exclusion of isolates in the analysis workflow. . 7

Supplementary Table 2. Demography of surveyed individuals and parasitological characteristics of *Plasmodium* spp. infections. 8

Supplementary Table 3. Proportions of isolates with positive PfHRP2 but negative detection by microscopy, based on their associated *18S rRNA* and *var*coding PCR detection status (N=46 isolates).......... 9

Supplementary Table 4. Proportions of *P. falciparum* detection by *var*coding (N=188 isolates). . 9

Supplementary Table 5. Microscopic detection and *P. falciparum* multiplicity of infection (Pf-MOI) categorised by host age group. 10

Supplementary Table 6. Number and proportions of *P. falciparum* infections with increased, decreased, or unchanged Pf-MOI from small to large pRBC volume, categorised by host age group. 11

Supplementary Figure 2. Agreement of Pf-MOI values for pairs of pRBC volumes sampled, indicated by Lin's concordance correlation coefficient (CCC) values. 12

Supplementary Figure 3. Directional genetic similarity between pairwise sampling of pRBC volumes, coloured by permutation of *P. falciparum*. 13

Supplementary Figure 4. Directional genetic similarity between pairwise sampling of pRBC volumes, coloured by *Plasmodium* spp. parasite density. 14

Supplementary Figure 5. Directional genetic similarity between pairwise sampling of pRBC volumes, coloured by age group of isolates (i.e. host). 15

Supplementary Figure 6. Analysis of repeat isolates in the context of varying pRBC volumes. . 16

Supplementary Figure 7. Comparison of Pf-MOI values for isolates with Pf-MOI_S = 0 OR Pf-MOI_L. 17

Supplementary Table 7. Data underlying comparisons in Supplementary Figure 7. 17

Supplementary Figure 8. Non-linear relationships between fold differences in Pf-MOI ($FD_{Pf\text{-}MOI}$, shown in log scale) and the Pf-MOI in smaller compared pRBC volumes (Pf-MOI_S). 18

Supplementary Table 8. Power calculation based on proportion of samples with Pf-MOI > 1. . 19

Supplementary Table 9. Estimated genetic similarity between isolate repertoires by pairwise type sharing (PTS) categorised by host age group and sampled pRBC volumes. 20

Supplementary Figure 9. Rarefaction curves of DBLα tags in the upsA and non-upsA groups. .. 20

Supplementary Figure 10. Proportion of DBLα types recovered from DBS and/or pRBC samples. 21

Supplementary Table 10. Proportion of DBLα types recovered from DBS and/or pRBC samples. 21

Supplementary Table 11. Statistical tests to examine association between cumulative complexity and host/spatial characteristics. 22

Supplementary Table 12. Adjusted prevalence of different *Plasmodium* spp. was used to predict the number of missed cases in the Bongo District and the Upper East Region in Ghana when using only DBS data. 23

Supplementary Table 13. Data underlying Figure 5a showing *Plasmodium* spp. infection prevalence (%) in Bongo located in the Upper East Region of Ghana, based on data from the Malaria Atlas Project (MAP) and estimates from this study. 24

Supplementary Table 14. Data underlying Figure 5b showing the impact of underestimated complexity on metrics that count infected hosts. 24

Supplementary Table 15. Data underlying Figure 5c showing the impact of underestimated complexity on metrics that count diverse parasites. 24

Supplementary Method 1. Estimation of whole blood (WB) and packed red blood cell (pRBC) volume equivalent in dried blood spots (DBS)

Blood samples were obtained with consent from a 34-year-old male (Hb 11.8 g/dL, RDT negative). First, we pipetted specific volumes of blood in triplicates (1, 2, 5, 10, 15, 20, 25, 30, 50µL) onto 3MM Whatman filter papers. We then let these DBS air dry as we would in the field for ~45min to 1 hour. We then measured the diameter of each DBS across two directions (*d1* and *d2*) and calculated the average diameter (*d*) for each DBS across the two measurements. The average radius (*r*) values were used to calculate the area (*A*) of DBS (Area, $A = \pi r^2$).

Measurements of DBS diameters and calculations of average DBS area

WB Volume (µL)	Repeat	DBS Diameter (d1, cm)	DBS Diameter (d2, cm)	Average DBS Diameter (d, cm)	Average DBS Radius (r, cm)	Average DBS Area (A, cm^2)
**1.00**	1	0.40	0.30	0.35	0.18	0.10
	2	0.30	0.30	0.30	0.15	0.07
	3	0.40	0.40	0.40	0.20	0.13
**2.00**	1	0.50	0.50	0.50	0.25	0.20
	2	0.40	0.50	0.45	0.23	0.16
	3	0.50	0.50	0.50	0.25	0.20
**5.00**	1	0.70	0.70	0.70	0.35	0.38
	2	0.80	0.70	0.75	0.38	0.44
	3	0.70	0.60	0.65	0.33	0.33
**10.00**	1	0.80	0.90	0.85	0.43	0.57
	2	0.80	0.80	0.80	0.40	0.50
	3	0.90	0.90	0.90	0.45	0.64
**15.00**	1	1.10	1.00	1.05	0.53	0.87
	2	1.00	1.00	1.00	0.50	0.79
	3	1.00	1.10	1.05	0.53	0.87
**20.00**	1	1.20	1.10	1.15	0.58	1.04
	2	1.10	1.20	1.15	0.58	1.04
	3	1.10	1.10	1.10	0.55	0.95
**25.00**	1	1.30	1.30	1.30	0.65	1.33
	2	1.20	1.20	1.20	0.60	1.13
	3	1.30	1.20	1.25	0.63	1.23
**30.00**	1	1.40	1.30	1.35	0.68	1.43
	2	1.30	1.20	1.25	0.63	1.23
	3	1.30	1.20	1.25	0.63	1.23
**50.00**	1	1.50	1.50	1.50	0.75	1.77
	2	1.50	1.40	1.45	0.73	1.65
	3	1.40	1.50	1.45	0.73	1.65

This resulted in a non-linear relationship between WB volume (µL) and the DBS area (cm^2), shown below.

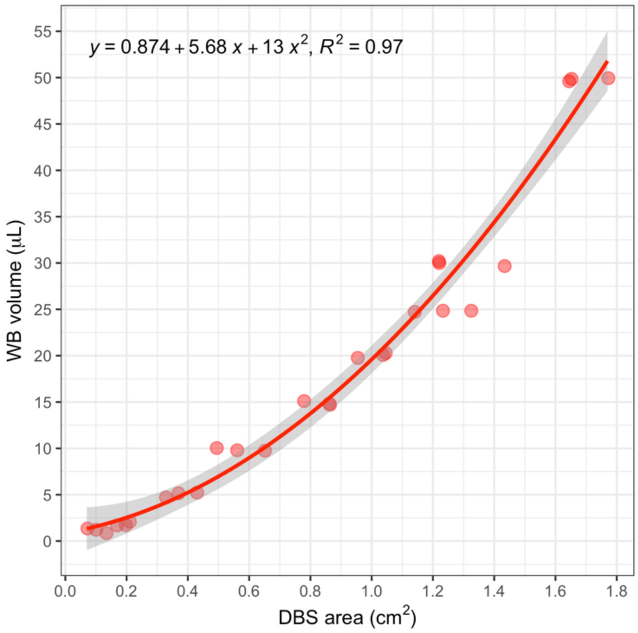

Relationship between WB volume on DBS and area of DBS

Based on this relationship, we estimated the volume of blood we obtain when we cut (based on area):

i. One 0.5cm × 0.5cm square (A = 1 × 0.25cm^2) → ~3.11µL of WB
ii. Two 0.5cm × 0.5cm squares (A = 2 × 0.25cm^2) → ~6.21µL of WB
iii. One 0.5cm × 1 cm rectangle (A = 1 × 0.50cm^2) → ~6.97µL of WB

DBS cuttings in our previous studies have used methods ii and iii[1,2], giving us an average of ~6-7µL equivalent of WB volume. Given the average expected proportion of RBCs at about 40%[3], this then translates into the equivalent of ~2.4-2.8µL packed red blood cells (pRBC).

Additional notes: We noted that, the DBS were larger when using larger volumes of blood, as was expected. However, more importantly, larger blood volumes tended to soak further through into the filter paper. In contrast, smaller volumes of blood expanded but did not soak through the filter as much compared to the larger blood volumes. This explains the non-linear relationship between WB volume collected on the DBS and the calculated DBS area.

Supplementary Method 2. Adjusting prevalence, Pf-MOI, and *P. falciparum* census population size with scale up to larger regions in Bongo and Upper East Ghana.

Calculating prevalence: We estimated prevalence of the different species based on DBS from 1,809 individuals living in Bongo collected at the time of this study. Prevalence was calculated by species (*P. falciparum*, *P. malariae*, *P. ovale* spp.) and age (6-9 years, 10-19 years, 20-39 years, ≥40 years). Prevalence levels were further adjusted based on the fold differences observed between DBS or 100µL in Figure 1, estimated in this study with *18S rRNA* with the same age stratification.

Estimating mean MOI and P. falciparum census population size: We first estimated Pf-MOI for 247 individuals living in Bongo. These individuals were microscopy-positive for *P. falciparum* with Pf-MOI ≥ 1 based on *var*coding of DBS samples. Using the GAM model fitted to the 1µL *vs* 100µL data, these DBS-estimated Pf-MOI values were further adjusted to predict Pf-MOI values that would be measured from deeper sampled volume. Mean MOI was calculated for both DBS and adjusted values over total sampled individuals without age stratification. Subsequently, this was used to calculate *P. falciparum* census population size[3]:

$$P.falciparum\ census\ pop\ size = \ mean\ MOI * host\ population\ size$$

In this study, we did not perform age-stratified calculations due to small sample sizes and limited statistical power for MOI correction within each age group. However, we emphasise the importance of conducting larger studies to estimate mean MOI and *P. falciparum* census population size separately for each age group and subsequently aggregate these estimates to determine the overall *P. falciparum* census population size. This approach is essential for accurately capturing host age-related variation in MOI.

Scaling up to larger population sizes: Prevalence, mean MOI, and *P. falciparum* census population size values were further scaled up to the larger population sizes of the Bongo District and the Upper East Region of Ghana to obtain an approximation of the number of infections or parasites underestimated in these regions.

Total and age-structured population size data was first accessed through the City Population website[22] based on the 2021 Population and Housing census by the Ghana Statistical Service (GSS)[23]. Population sizes in Ghana:

- Bongo District: 120,254 [total]; 30,416 [0-9 years]; 28,494 [10-19 years]; 35,135 [20-39 years]; 26,209 [≥40 years].
- Upper East Region: 1,301,226 [total]; 334,250 [0-9 years]; 304,105 [10-19 years]; 378,958 [20-39 years]; 283,913 [≥40 years].

Given the exclusion of children ≤ 5 years from this study, we further split the population size data for 0-9 years with a 6:4 ratio into two age groups 0-5 years and 6-9 years. Relevant to our study. The final age-structured population size data as follows:

- Bongo District: 102,004 [≥6 years]; 12,166 [6-9 years]; 28,494 [10-19 years]; 35,135 [20-39 years]; 26,209 [≥40 years].
- Upper East Region: 1,100,676 [≥6 years]; 133,700 [6-9 years]; 304,105 [10-19 years]; 378,958 [20-39 years]; 283,913 [≥40 years].

Map of regions and districts in Ghana in Figure 4a was drawn with Global ADMinistrative area (GADM) data downloaded for Ghana (v4.1)[24]. From the Malaria Atlas Project (MAP)[25,26], we downloaded data on 'Infection Prevalence' standardised based on the proportion of children aged 2-10 years in Ghana in 2020 (also the year of our field sampling), shown in Figure 5a. In Figure 5b, infection prevalence in children aged 2-10 years in Bongo is shown (pixel location: 'Bongo, Upper East, Ghana').

Supplementary Figure 1. Microscopic *P. falciparum* densities were significantly lower in asymptomatic isolates compared to clinical isolates during the same epidemiological survey. Parasite densities in clinical isolates are ~55x that of asymptomatic isolates, with median densities of 29,600 parasites/µL (*N*=93, IQR: 15,720-44,000) and 520 parasites/µL (*N*=295, IQR: 240-2,120) for clinical and asymptomatic, respectively. Distributions are significantly different by Wilcoxon rank sum test (*p-value < 0.001*).

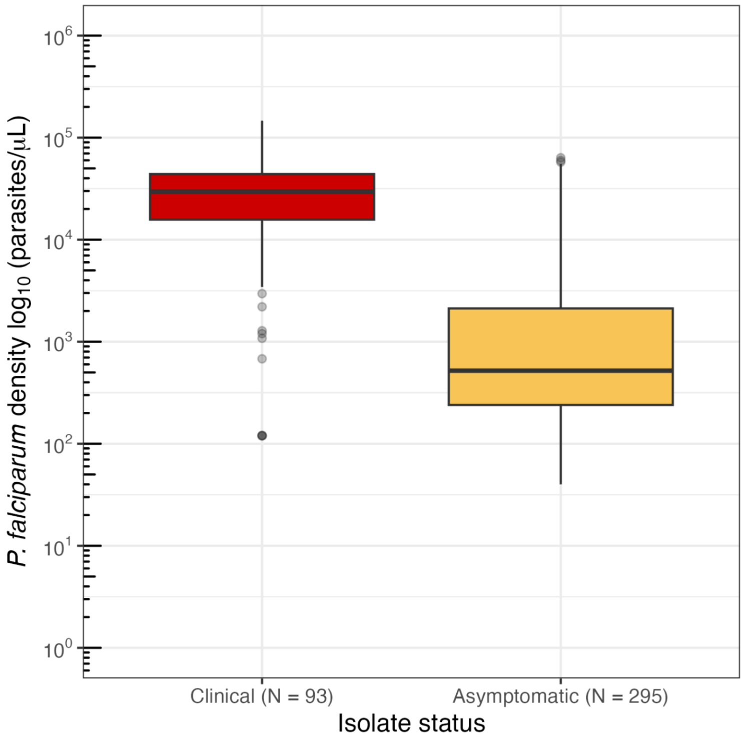

Supplementary Table 1. Dataset sizes following exclusion of isolates in the analysis workflow.

Parameters	Remaining number of isolates
**I.** Isolates sampled for pRBC	200
**II.** Isolates with no pRBC duplicates	193 (96.5%)*
**III.** Isolates with no DBS duplicates	192 (96.0%)*
**IV.** Isolates with matching epidemiological data	191 (95.5%)*
**V.** Isolates found to be not symptomatic	188 (94.0%)*
**VI.** Isolates with repertoire size ≥ 1 DBLα types in any of the four pRBC volume (*var*coding)^	147 (78.2%)
1μL+	105 (71.4%)
10μL+	119 (81.0%)
50μL+	131 (89.1%)
100μL+	142 (96.6%)
**VII.** Isolates with repertoire size ≥ 20 DBLα types in any of the four pRBC volume (*var*coding)^	113 (60.1%)
1μL+	55 (48.7%)
10μL+	91 (80.5%)
50μL+	106 (93.8%)
100μL+	113 (100.0%)

* Data reflect No. (% [n/N]) of 200 isolates sampled in (I).
^ Data reflect No. (% [n/N]) of remaining isolates in (V).
+ Data reflect No. (% [n/N]) of remaining isolates in (VI or VII).

Supplementary Table 2. Demography of surveyed individuals and parasitological characteristics of *Plasmodium* spp. infections.

Characteristic		Sample size*	
**All**		188	
**Age groups**	6-10 years	46 (24.5%)	
	11-20 years	47 (25.0%)	
	21-39 years	44 (23.4%)	
	≥40 years	51 (27.1%)	
**Sex**	Female	100 (53.2%)	
	Male	88 (46.8%)	
**Village**	Vea	89 (47.3%)	
	Gowrie	99 (52.7%)	
		**Microscopy-positive^**	***Plasmodium* spp. median density†**
**All**		33 (17.6%)	720 [320-2880]
**Age groups**	6-10 years	9 (27.3%)	840 [360-3720]
	11-20 years	12 (36.4%)	1,100 [490-5760]
	21-39 years	4 (12.1%)	240 [130-330]
	≥40 years	8 (24.2%)	520 [120-1600]
**Sex**	Female	22 (66.7%)	520 [320-3900]
	Male	11 (33.3%)	960 [280-1800]
**Village**	Vea	16 (48.5%)	520 [240-1880]
	Gowrie	17 (51.5%)	900 [320-3850]

Abbreviations: IQR, interquartile range
* Data reflect No. (% [n/N]) of individuals sampled.
^ Data reflect No. (% [n/N]) of individuals sampled with isolates that were microscopically-positive for *Plasmodium* spp., predominantly infected with *P. falciparum*, including mixed infections.
† Median parasite density for the microscopically-positive *Plasmodium* spp. isolates, predominantly *P. falciparum* isolates, including mixed infections (parasites/µl [IQR]).

Supplementary Table 3. Proportions of isolates with positive PfHRP2 but negative detection by microscopy, based on their associated *18S rRNA* and *var*coding PCR detection status (N=46 isolates).

	Number of Isolates	Proportion of Isolates
**Positive based on species-specific *18S rRNA* PCR**		
DBS	27	58.7%
100µL-pRBC	38	82.6%
**Positive based on *var*coding PCR**		
1µL-pRBC	28	60.9%
10µL-pRBC	34	73.9%
50µL-pRBC	39	84.8%
100µL-pRBC	43	93.5%

Supplementary Table 4. Proportions of *P. falciparum* detection by *var*coding (N=188 isolates). Detection is shown for the four pRBC volumes at a minimum isolate repertoire size thresholds of 1 and 20 DBLα types. E.g. "N-Y-Y-Y" indicates positive detection in 10, 50, and 100µL volumes only.

Detection code (1-10-50-100µL)	Minimum isolate repertoire size	Number of Isolates	Proportion of Isolates
N-N-N-N	1	41	21.8%
N-N-N-Y	1	12	6.4%
N-N-Y-N	1	2	1.1%
N-N-Y-Y	1	11	5.9%
N-Y-N-Y	1	2	1.1%
N-Y-Y-N	1	2	1.1%
N-Y-Y-Y	1	13	6.9%
Y-N-N-Y	1	2	1.1%
Y-N-Y-Y	1	1	0.5%
Y-Y-Y-N	1	1	0.5%
Y-Y-Y-Y	1	101	53.7%

Supplementary Table 5. Microscopic detection and *P. falciparum* multiplicity of infection (Pf-MOI) categorised by host age group. Data reflect No. (% [n/N]) of isolates in each category. Pf-MOI ≥ 1 defined for isolates with repertoire size ≥ 1.

Host age group (years)	MOI	TOTAL				Microscopy negative (-)				Microscopy positive (+)			
		1µL	10µL	50µL	100µL	1µL	10µL	50µL	100µL	1µL	10µL	50µL	100µL
All	0	83 (44.1)	69 (36.7)	57 (30.3)	46 (24.5)	82 (52.9)	69 (44.5)	57 (36.8)	46 (29.7)	1 (3.0)	0 (0.0)	0 (0.0)	0 (0.0)
	1	69 (36.7)	44 (23.4)	39 (20.7)	45 (23.9)	60 (38.7)	41 (26.5)	36 (23.2)	43 (27.7)	9 (27.3)	3 (9.1)	3 (9.1)	2 (6.1)
	≥ 2	36 (19.1)	75 (39.9)	92 (48.9)	97 (51.6)	13 (8.4)	45 (29.0)	62 (40.0)	66 (42.6)	23 (69.7)	30 (90.9)	30 (90.9)	31 (93.9)
**TOTAL**		**188**				**155**				**33**			
6-10	0	21 (45.7)	18 (39.1)	16 (34.8)	9 (19.6)	20 (54.1)	18 (48.6)	16 (43.2)	9 (24.3)	1 (11.1)	0 (0.0)	0 (0.0)	0 (0.0)
	1	11 (23.9)	8 (17.4)	9 (19.6)	13 (28.3)	10 (27.0)	7 (18.9)	7 (18.9)	12 (32.4)	1 (11.1)	1 (11.1)	2 (22.2)	1 (11.1)
	≥ 2	14 (30.4)	20 (43.5)	21 (45.7)	24 (52.2)	7 (18.9)	12 (32.4)	14 (37.8)	16 (43.2)	7 (77.8)	8 (88.9)	7 (77.8)	8 (88.9)
**TOTAL**		**46**				**37**				**9**			
11-20	0	17 (36.2)	17 (36.2)	16 (34.0)	9 (19.1)	17 (48.6)	17 (48.6)	16 (45.7)	9 (25.7)	0 (0.0)	0 (0.0)	0 (0.0)	0 (0.0)
	1	17 (36.2)	7 (14.9)	4 (8.5)	12 (25.5)	15 (42.9)	7 (20.0)	4 (11.4)	12 (34.3)	2 (16.7)	0 (0.0)	0 (0.0)	0 (0.0)
	≥ 2	13 (27.7)	23 (48.9)	27 (57.4)	26 (55.3)	3 (8.6)	11 (31.4)	15 (42.9)	14 (40.0)	10 (83.3)	12 (100.0)	12 (100.0)	12 (100.0)
**TOTAL**		**47**				**35**				**12**			
21-39	0	23 (52.3)	18 (40.9)	14 (31.8)	15 (34.1)	23 (57.5)	18 (45.0)	14 (35.0)	15 (37.5)	0 (0.0)	0 (0.0)	0 (0.0)	0 (0.0)
	1	17 (38.6)	11 (25.0)	13 (29.5)	10 (22.7)	15 (37.5)	11 (27.5)	13 (32.5)	10 (25.0)	2 (50.0)	0 (0.0)	0 (0.0)	0 (0.0)
	≥ 2	4 (9.1)	15 (34.1)	17 (38.6)	19 (43.2)	2 (5.0)	11 (27.5)	13 (32.5)	15 (37.5)	2 (50.0)	4 (100.0)	4 (100.0)	4 (100.0)
**TOTAL**		**44**				**40**				**4**			
≥40	0	22 (43.1)	16 (31.4)	11 (21.6)	13 (25.5)	22 (51.2)	16 (37.2)	11 (25.6)	13 (30.2)	0 (0.0)	0 (0.0)	0 (0.0)	0 (0.0)
	1	24 (47.1)	18 (35.3)	13 (25.5)	10 (19.6)	20 (46.5)	16 (37.2)	12 (27.9)	9 (20.9)	4 (50.0)	2 (25.0)	1 (12.5)	1 (12.5)
	≥ 2	5 (9.8)	17 (33.3)	27 (52.9)	28 (54.9)	1 (2.3)	11 (25.6)	20 (46.5)	21 (48.8)	4 (50.0)	6 (75.0)	7 (87.5)	7 (87.5)
**TOTAL**		**51**				**43**				**8**			

Supplementary Table 6. Number and proportions of *P. falciparum* infections with increased, decreased, or unchanged Pf-MOI from small to large pRBC volume, categorised by host age group. Data reflect No. (% [n/N]) of isolates in each category. Pf-MOI ≥ 1 defined for isolates with repertoire size ≥ 1.

pRBC Volumes Compared	Age Group	Decreased	Unchanged	Increased
**1µL to 10µL**	6-10 Years	2 (6.90)	9 (31.03)	18 (62.07)
	11-20 Years	1 (3.23)	10 (32.26)	20 (64.52)
	21-39 Years	0 (0.00)	7 (26.92)	19 (73.08)
	40+ Years	1 (2.78)	14 (38.89)	21 (58.33)
**1µL to 50µL**	6-10 Years	1 (3.23)	7 (22.58)	23 (74.19)
	11-20 Years	1 (3.13)	4 (12.50)	27 (84.38)
	21-39 Years	0 (0.00)	4 (13.33)	26 (86.67)
	40+ Years	0 (0.00)	7 (17.50)	33 (82.50)
**1µL to 100µL**	6-10 Years	0 (0.00)	6 (16.22)	31 (83.78)
	11-20 Years	0 (0.00)	6 (15.79)	32 (84.21)
	21-39 Years	0 (0.00)	4 (13.79)	25 (86.21)
	40+ Years	1 (2.56)	4 (10.26)	34 (87.18)
**10µL to 50µL**	6-10 Years	3 (9.38)	13 (40.63)	16 (50.00)
	11-20 Years	2 (6.45)	13 (41.94)	16 (51.61)
	21-39 Years	1 (3.33)	13 (43.33)	16 (53.33)
	40+ Years	0 (0.00)	16 (40.00)	24 (60.00)
**50µL to 100µL**	6-10 Years	4 (10.81)	18 (48.65)	15 (40.54)
	11-20 Years	5 (13.16)	14 (36.84)	19 (50.00)
	21-39 Years	4 (13.33)	20 (66.67)	6 (20.00)
	40+ Years	9 (21.43)	19 (45.24)	14 (33.33)

Supplementary Figure 2. Agreement of Pf-MOI values for pairs of pRBC volumes sampled, indicated by Lin's concordance correlation coefficient (CCC) values. Plots are shown for isolates with Pf-MOI ≥ 1 (and with isolate repertoire size ≥ 1) for all four pRBC volume. Pf-MOI_S and Pf-MOI_L represent *P. falciparum* MOI of the smaller and larger pRBC volumes, respectively, in a pairwise comparison. The strongest concordance in Pf-MOI was observed for 50µL and 100µL pRBC volumes whereas the lowest concordance in Pf-MOI was observed for 1µL and 100µL pRBC volumes.

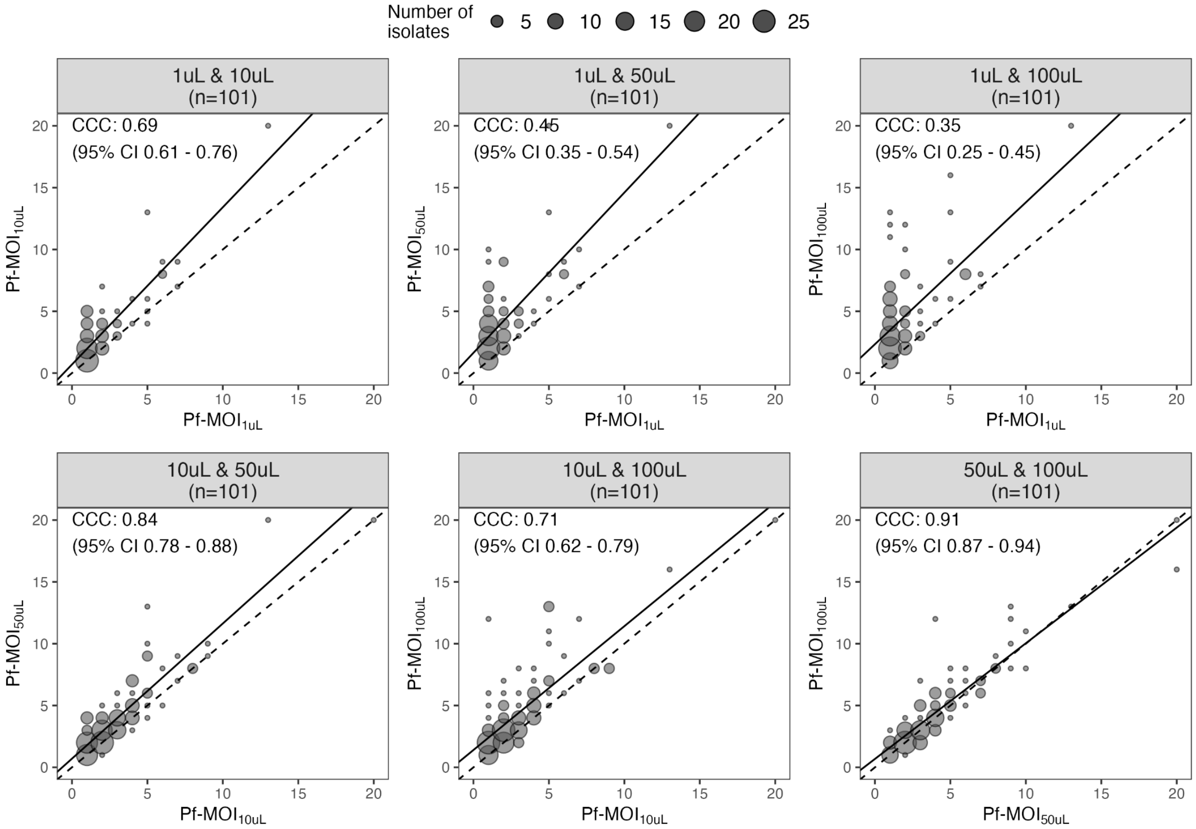

Supplementary Figure 3. Directional genetic similarity between pairwise sampling of pRBC volumes, coloured by permutation of *P. falciparum*. Plots are shown for isolates with Pf-MOI ≥ 1 for all four pRBC volumes (and with isolate repertoire size ≥ 20). PTS_S and PTS_L represent directional genetic similarity levels calculated relative to repertoire sizes of the smaller and larger pRBC volumes, respectively, in a pairwise comparison. **(a)** Each data point represents an isolate, coloured by its permutation of *P. falciparum* detection in the four pRBC volumes. **(b)** Distributions of directional PTS values are coloured by the smaller volume (Vol_S).

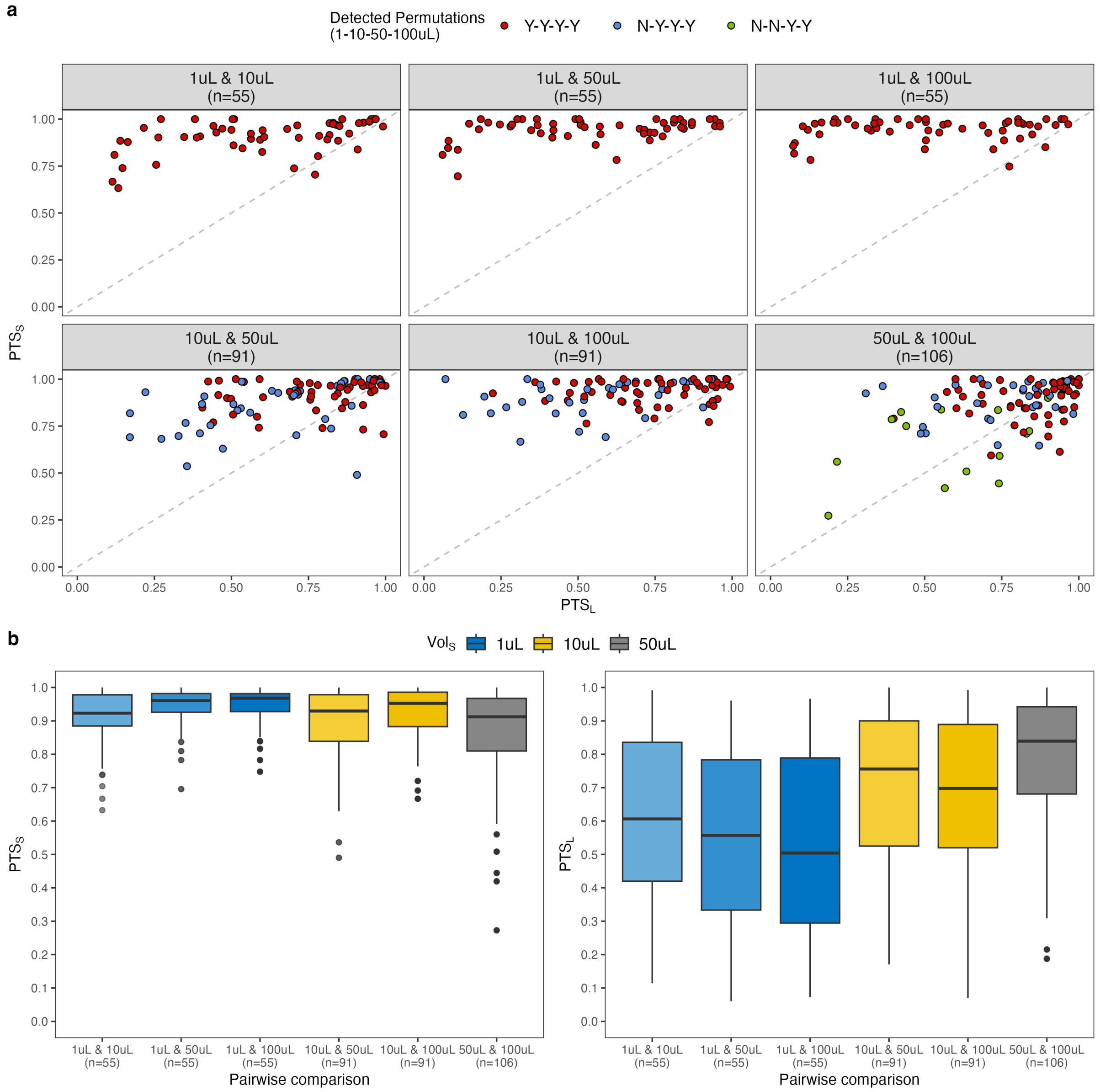

Supplementary Figure 4. Directional genetic similarity between pairwise sampling of pRBC volumes, coloured by *Plasmodium* spp. parasite density. Scatterplots are shown for isolates with Pf-MOI ≥ 1 for all four pRBC volumes (and with isolate repertoire size ≥ 20). Each row of panels represent a parasite density bin at 0 (or undetectable, <40 parasites/µL), 40-999 parasites/µL, 1,000-9,999 parasites/µL, and ≥10,000 parasites/µL). PTS_S and PTS_L represent directional genetic similarity levels calculated relative to repertoire sizes of the smaller (S) and larger (L) pRBC volumes, respectively, in a pairwise comparison. Each data point represents an isolate. Isolates without detectable parasite density information are coloured in grey.

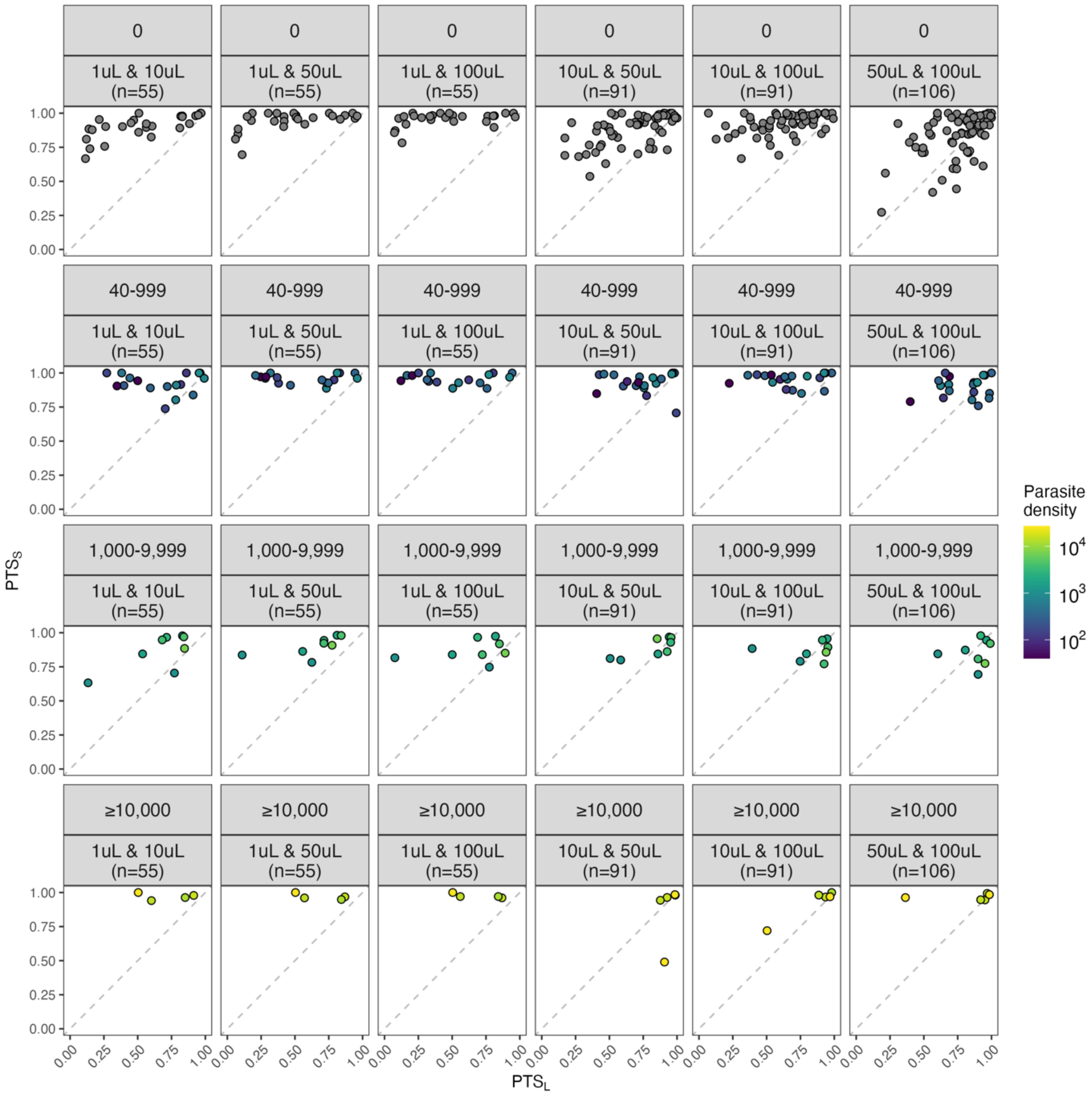

Supplementary Figure 5. Directional genetic similarity between pairwise sampling of pRBC volumes, coloured by age group of isolates (i.e. host). Scatterplots are shown for isolates with Pf-MOI ≥ 1 for all four pRBC volumes (and with isolate repertoire size ≥ 20). Each data point represents an isolate.

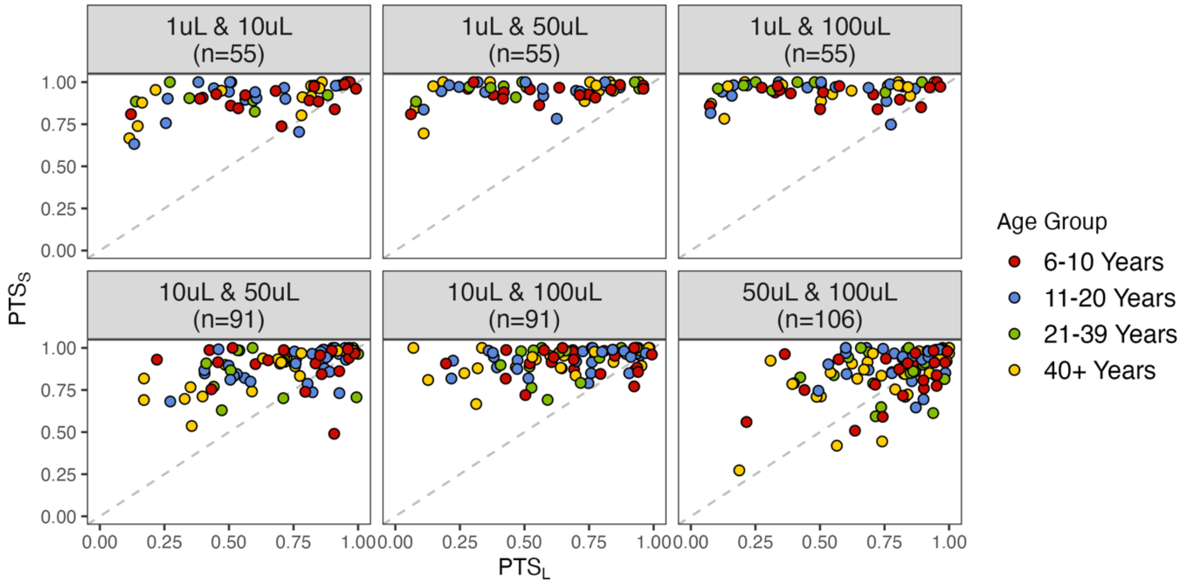

Supplementary Figure 6. Analysis of repeat isolates in the context of varying pRBC volumes. Here, "R1" represents 40 field isolates included in the initial dataset while "R2" represents the repeat of these isolates. **(a)** Estimated Pf-MOI for the repeat of every isolate, represented on the y-axis. Both the initial (R1) and repeat (R2) isolates confirmed the volume-based observations reported in this study, showing increased detection of *P. falciparum* and Pf-MOI for both R1 and R2 sets of isolates as larger pRBC volumes were sampled. **(b)** Comparison of genetic similarity by PTS between repeats at every pRBC volume. PTS values were lowest for repeats of 1µL pRBC volumes and highest for the largest 100µL pRBC volume, suggesting that sampling consistency was greater when done in larger volumes. **(c)** Based on Lin's concordance correlation coefficient (CCC), there was strong concordance in estimated Pf-MOI between repeats of all pRBC volumes, particularly in larger pRBC volumes of 50µL or 100µL. **(d)** Distributions of isolate repertoire size and Pf-MOI showed no significant differences between repeats (adjusted p-value > 0.05). Statistical analysis was conducted with the pairwise Wilcoxon signed rank tests. Levels of significance in pairwise tests were adjusted using the "holm" correction. Median Pf-MOI for both R1 and R2 increased from 1 to 2 comparing the 1µL & 100µL pRBC datasets.

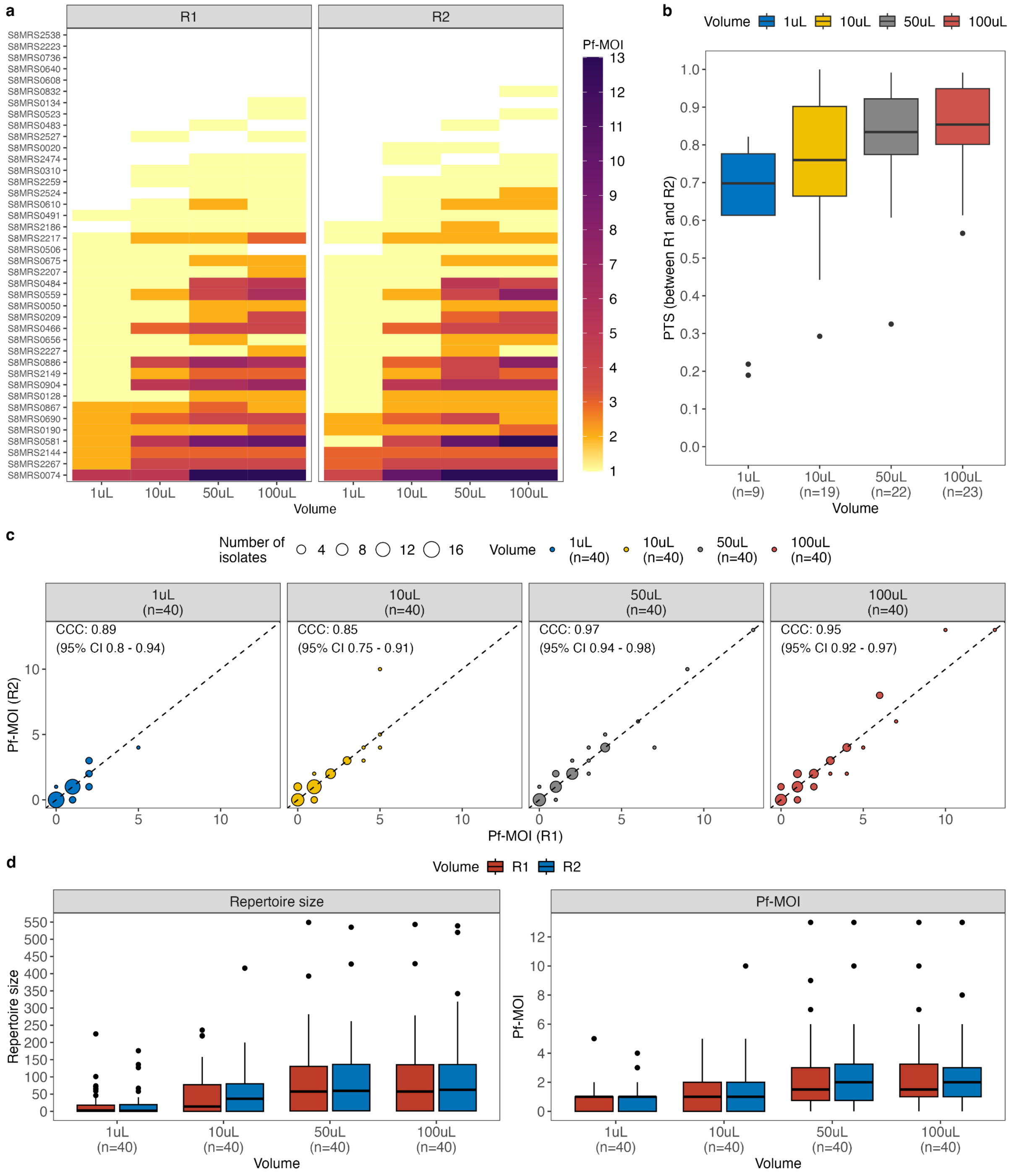

Supplementary Figure 7. Comparison of Pf-MOI values for isolates with Pf-MOI$_S$ = 0 OR Pf-MOI$_L$. Pf-MOI$_S$ and Pf-MOI$_L$ represent the Pf-MOI values of the smaller and larger compared pRBC volumes, respectively. This figure explores comparisons where, if an isolate has either Pf-MOI$_S$ = 0 OR Pf-MOI$_L$ = 0, what is the Pf-MOI of the corresponding pair value. For such isolates, the estimated Pf-MOI for two pRBC volumes mostly differed by one. Data underlying this figure is shown in Supplementary Table 7.

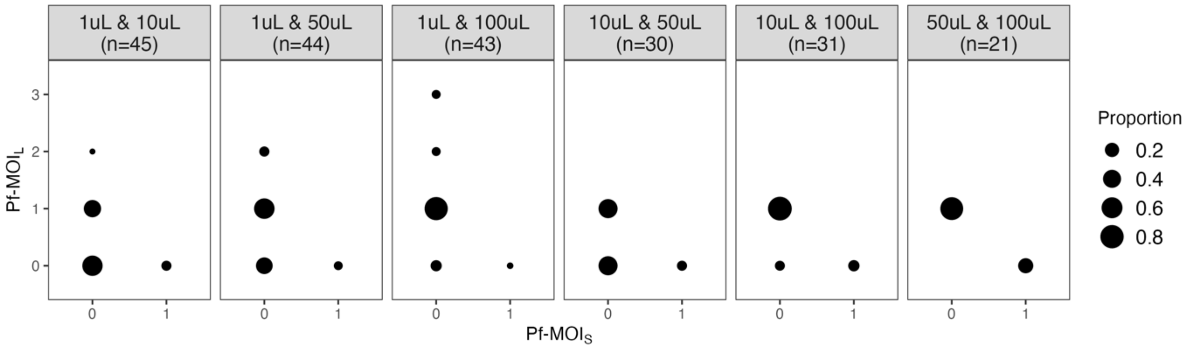

Supplementary Table 7. Data underlying comparisons in Supplementary Figure 7.

	pRBC volume pairs	Pf-MOI$_S$	Pf-MOI$_L$	Number of Isolates	Proportion of Isolates (Pf-MOI$_S$ or Pf-MOI$_L$ = 0)
when Pf-MOI$_S$=0	1µL & 10µL	0	0	25	59.5%
		0	1	16	38.1%
		0	2	1	2.4%
	1µL & 50µL	0	0	14	33.3%
		0	1	25	59.5%
		0	2	3	7.2%
	1µL & 100µL	0	0	4	9.5%
		0	1	34	81.0%
		0	2	2	4.8%
		0	3	2	4.8%
	10µL & 50µL	0	0	14	50.0%
		0	1	14	50.0%
	10µL & 100µL	0	0	2	7.1%
		0	1	26	92.9%
	50µL & 100µL	0	1	16	100.0%
when Pf-MOI$_L$=0	1µL & 10µL	0	0	25	89.3%
		1	0	3	10.7%
	1µL & 50µL	0	0	14	87.5%
		1	0	2	12.5%
	1µL & 100µL	0	0	4	80.0%
		1	0	1	20.0%
	10µL & 50µL	0	0	14	87.5%
		1	0	2	12.5%
	10µL & 100µL	0	0	2	40.0%
		1	0	3	60.0%
	50µL & 100µL	1	0	5	100.0%

Supplementary Figure 8. Non-linear relationships between fold differences in Pf-MOI ($FD_{Pf\text{-}MOI}$, shown in log scale) and the Pf-MOI in smaller compared pRBC volumes ($Pf\text{-}MOI_S$). Scatterplots are shown for isolates with Pf-MOI ≥ 1 (and with isolate repertoire size ≥ 1) for all four pRBC volumes. $FD_{Pf\text{-}MOI}$ is shown in log scale on the y-axis. Horizontal red, dashed line at value of 2^0 indicates no difference in estimated Pf-MOI ($FD_{Pf\text{-}MOI} = 2^0 = 1$). Green solid lines show fitted smooth curves, grey background indicates the 95% CI.

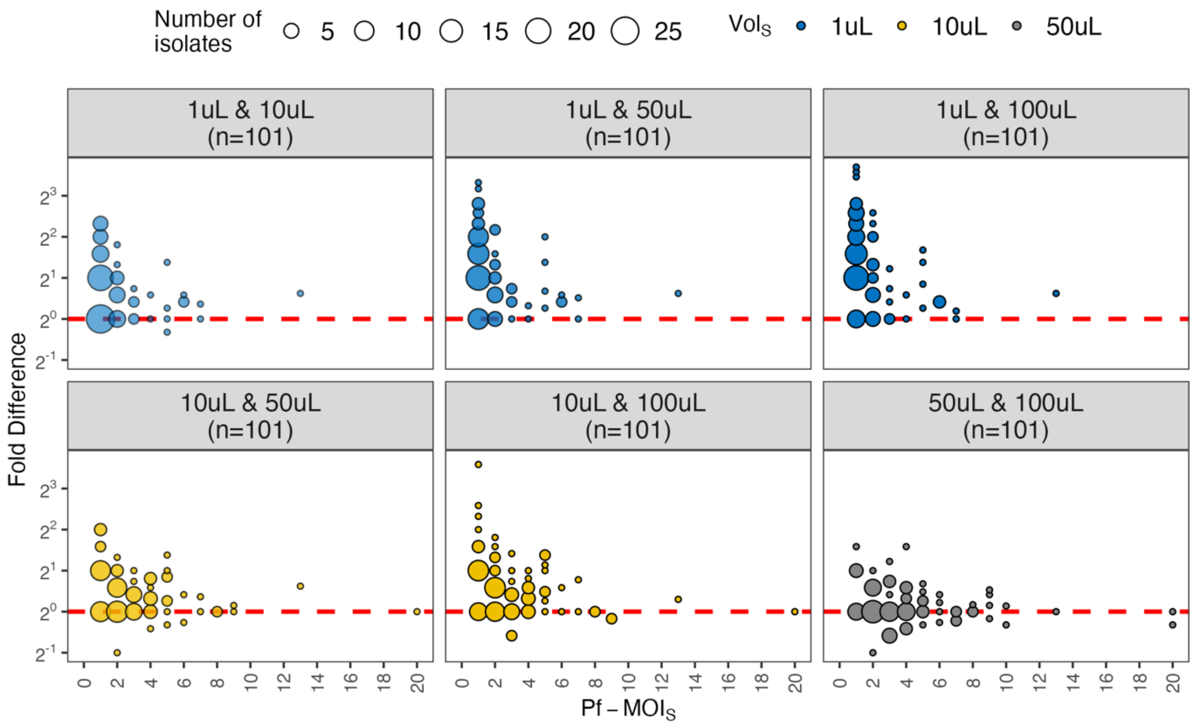

Supplementary Table 8. Power calculation based on proportion of samples with Pf-MOI > 1. This estimates statistical power (p) of the current dataset to provide correction scales between 1µL and larger pRBC volumes (α = 0.05). This was then followed by estimations of sample sizes (n) required to achieve sufficient power (power ≥ 0.80, α = 0.05) for correction scales stratified by host age group.

CURRENT DATASET									Sample size (n) required to achieve p ≥ 0.80
**Host age group (years)**	**pRBC vol 1**	**pRBC vol 2**	**n1[+] (vol1)**	**n2[+] (vol2)**	**% Pf-MOI>1[^] (vol1)**	**% Pf-MOI>1[^] (vol2)**	**Effect size (h)**	**Statistical power**	
All	10µL	1µL	101	101	66.7	32.5	0.78	1.00	
	50µL	1µL	101	101	81.2	32.5	1.16	1.00	
	100µL	1µL	101	101	85.5	32.5	1.29	1.00	
6-10	10µL	1µL	24	24	74.2	51.6	0.56	0.50[#]	50
	50µL	1µL	24	24	77.4	51.6	0.68	0.65[#]	34
	100µL	1µL	24	24	87	51.6	0.99	0.93	16
11-20	10µL	1µL	29	29	82.1	46.4	0.73	0.79[#]	30
	50µL	1µL	29	29	96.4	46.4	1.14	0.99	13
	100µL	1µL	29	29	92.9	46.4	1.02	0.97	16
21-39	10µL	1µL	21	21	62.5	16.7	1.11	0.95	13
	50µL	1µL	21	21	70.8	16.7	1.34	0.99	9
	100µL	1µL	21	21	79.2	16.7	1.46	1.00	8
≥40	10µL	1µL	27	27	50	14.7	0.87	0.89	21
	50µL	1µL	27	27	79.4	14.7	1.57	1.00	7
	100µL	1µL	27	27	82.4	14.7	1.86	1.00	5

[+] Sample sizes available for two compared pRBC volumes.

[^] Proportion of samples with MOI > 1 (%).

[#] Comparisons where power < 0.80 at a significance level (α) of 0.05.

Supplementary Table 9. Estimated genetic similarity between isolate repertoires by pairwise type sharing (PTS) categorised by host age group and sampled pRBC volumes. These estimates were based on isolates with Pf-MOI ≥ 1 (and with isolate repertoire size ≥ 20).

**Population metrics**	**1μL**	**10μL**	**50μL**	**100μL**
Number of isolates	55	91	106	113
DBLα type richness	4,682	8,486	10,760	11,715
upsA	611	903	1,062	1,126
non-upsA	4,071	7,583	9,698	10,589
Pairwise type sharing				
Minimum	0.000	0.000	0.000	0.000
Maximum	0.124	0.301	0.400	0.365
Mean	0.018	0.026	0.028	0.027
Median	0.017	0.024	0.026	0.026
Q1	0.000	0.014	0.016	0.015
Q3	0.027	0.035	0.038	0.038

Supplementary Figure 9. Rarefaction curves of DBLα tags in the upsA and non-upsA groups. These estimates were based on isolates with Pf-MOI ≥ 1 (and with isolate repertoire size ≥ 20).

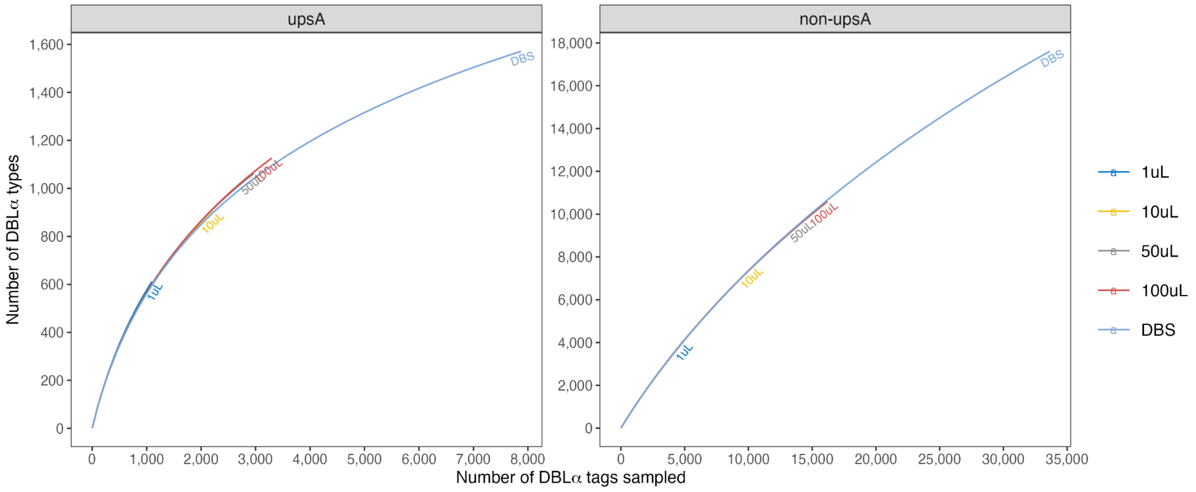

Supplementary Figure 10. Proportion of DBLα types recovered from DBS and/or pRBC samples. These estimates were based on isolates with Pf-MOI ≥ 1 (and with isolate repertoire size ≥ 20). This analysis included DBS samples (263 isolates) and pRBC samples (55, 91, 106, and 113 isolates for 1µL, 10µL, 50µL, and 100µL volumes, respectively). Each data point represents the proportion of DBLα types in the reservoir identified in isolates with specific combinations of DBS and pRBC volume (values in Supplementary Table 9). E.g. for 100µL pRBC, of the 21,222 DBLα types identified in 263 DBS and 113 pRBC samples, 32.1% was present in both "**DBS & pRBC**", 23.1% in "**pRBC only**", and the remainder of 44.8% in "**DBS only**". Finding subsets of DBLα types exclusively from different sample sources suggests that both sampling approaches are necessary and complementary to more accurately reflect true population-level metrices.

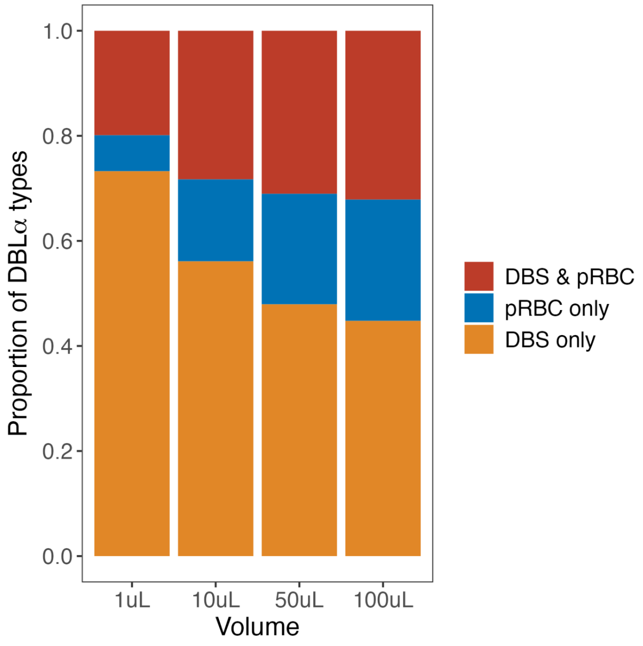

Supplementary Table 10. Proportion of DBLα types recovered from DBS and/or pRBC samples. These estimates were based on isolates with Pf-MOI ≥ 1 (and with isolate repertoire size ≥ 20).

pRBC volume	Combination	Number of DBLα types	Proportion of DBLα types (%)
1µL	DBS & pRBC	3,486	19.9%
	pRBC only	1,196	6.8%
	DBS only	12,842	73.3%
10µL	DBS & pRBC	5,472	28.3%
	pRBC only	3,014	15.6%
	DBS only	10,856	56.1%
50µL	DBS & pRBC	6,415	31.0%
	pRBC only	4,345	21.0%
	DBS only	9,913	48.0%
100µL	DBS & pRBC	6,821	32.1%
	pRBC only	4,894	23.1%
	DBS only	9,507	44.8%

Supplementary Table 11. Statistical tests to examine association between cumulative complexity and host/spatial characteristics. Human host characteristics include sex (male/female), age (years), haemoglobin levels, axillary temperature (°C), and occupation. Spatial characteristics include village (Vea/Gowrie) and sections.

Parameters	p-value	Test Statistic	Estimate	Statistical test
Host characteristics				
Sex	0.436	W = 2434	NA	Wilcoxon Rank Sum Test
Age	0.421	S = 542,268.42	-0.067 (rho)	Spearman Rank Correlation Test
Haemoglobin levels	0.199	S = 453,552.77	0.107 (rho)	Spearman Rank Correlation Test
Axillary temperature	0.644	S = 478,337.70	0.039 (rho)	Spearman Rank Correlation Test
Occupation	0.843	χ^2 = 2.72	NA	Kruskal-Wallis Rank Sum Test
Spatial characteristics				
Village	0.773	W = 2,535	NA	Wilcoxon Rank Sum Test
Section	0.950	χ^2 = 4.57	NA	Kruskal-Wallis Rank Sum Test

Supplementary Table 12. Adjusted prevalence of different *Plasmodium* spp. was used to predict the number of missed cases in the Bongo District and the Upper East Region in Ghana when using only DBS data. Fold difference in the observed prevalence from DBS and 100µL in this study was first estimated, i.e. "× difference". Infection prevalence was then estimated from DBS data from a larger surveyed group of *N*=1,809 individuals living in Bongo who were sampled at the same time, i.e. "Survey DBS - Bongo", stratified by age groups of 6-9 years (*n*=230), 10-19 years (*n*=563), 20-39 years (*n*=271), and ≥40 years (*n*=391). This was further adjusted through multiplication with the "× difference" factor to obtain prevalence in Bongo with deeper sampling, i.e. "Adjusted". Infection prevalence was then used to estimate the number of *Plasmodium* spp. infections in the Bongo District and the Upper East Region of Ghana, based on total and age-structured population size data accessed through the City Population website based on the 2021 Population and Housing census by the Ghana Statistical Service (GSS).

Age Groups (as per City Population)	Prevalence (%) (This Study, *N*=188)			Number of infections (Survey DBS, *N*=1,455)		Prevalence (Survey DBS, *N*=1,455)		Number of infections (Bongo, *N*=102,004)		Number of infections (Upper East, *N*=1,100,676)	
	DBS	100µL	× difference	DBS	Adjusted	DBS	Adjusted	DBS	Adjusted	DBS	Adjusted
P. falciparum											
All ages (≥6 years)	55.85	73.94	1.32								
6-9 years	40.63	62.50	1.54	107	165	46.52	71.57	5,660	8,707	62,200	95,692
10-19 years	62.07	81.03	1.31	381	497	67.67	88.35	19,283	25,175	205,798	268,680
20-39 years	46.81	65.96	1.41	147	207	54.24	76.43	19,058	26,855	205,560	289,653
≥40 years	66.67	80.39	1.21	213	257	54.48	65.69	14,278	17,217	154,664	186,506
P. malariae											
All ages (≥6 years)	7.45	14.36	1.93								
6-9 years	6.25	9.38	1.50	11	16	4.78	7.17	582	873	6,394	9,592
10-19 years	15.52	27.59	1.78	106	188	18.83	33.47	5,365	9,537	57,256	101,788
20-39 years	2.13	8.51	4.00	5	20	1.85	7.38	648	2,593	6,992	27,967
≥40 years	3.92	7.84	2.00	11	22	2.81	5.63	737	1,475	7,987	15,975
***P. ovale* spp.**											
All ages (≥6 years)	2.13	5.32	2.50								
6-9 years	3.13	9.38	3.00	5	15	2.17	6.52	264	793	2,907	8,720
10-19 years	3.45	6.90	2.00	31	62	5.51	11.01	1,569	3,138	16,745	33,489
20-39 years	0.00	2.13	3.13	3	9	1.11	3.46	389	1,216	4,195	13,121
≥40 years	1.96	3.92	2.00	8	16	2.05	4.09	536	1,072	5,809	11,618
P. vivax											
All ages (≥6 years)	0.00	0.00	0.00	0	0	0.00	0.00	0	0	0	0

Supplementary Table 13. Data underlying Figure 5a showing *Plasmodium* spp. infection prevalence (%) in Bongo located in the Upper East Region of Ghana, based on data from the Malaria Atlas Project (MAP) and estimates from this study. Comparison of *P. falciparum* prevalences in Bongo in year 2020 estimated by MAP in children 2-10 years ($PfPR_{2-10}$ data for year 2020), by *18S rRNA* in our larger DBS survey in children 2-10 years, in our larger DBS survey in all ages, and adjusted for larger blood volume sampling (100µL-pRBC) in all ages. Also shown are prevalences for minor species *P. malariae* and *P. ovale* spp. that are not available in MAP.

	P. falciparum	***P. malariae***	***P. ovale* spp.**
Malaria Atlas Project (2-10yr)	8.90	*NA*	*NA*
Survey DBS (2-10yr)	34.64	3.91	1.49
Survey DBS (all ages)	49.53	7.35	2.60
Survey DBS (all ages, and adjusted for 100µL-pRBC sampling)	65.57	14.18	6.50

Supplementary Table 14. Data underlying Figure 5b showing the impact of underestimated complexity on metrics that count infected hosts. In this table, "Adjusted" represents adjusted estimates to account for deeper volume sampling (i.e. 100µL). Prevalence and adjusted prevalence estimated from the larger DBS survey in individuals ≥6 years were scaled up to approximate the number of malaria cases in the Bongo DBS survey, Bongo District, and the Upper East Region of Ghana. Similarly, the numbers of heavily-infected individuals with extreme metagenomic complexity in these regions were approximated with the 5.85% prevalence reported in this study.

Counting Infected Hosts		**Survey in Bongo (1,455 individuals)**		**Scale up for Bongo District (102,004 individuals)**		**Scale up for Upper East Ghana (1,100,676 individuals)**	
		**DBS**	**Adjusted**	**DBS**	**Adjusted**	**DBS**	**Adjusted**
Number of cases	*P. falciparum*	848	1,126	58,279	77,954	628,222	840,531
	P. malariae	133	246	7,332	14,478	78,629	155,322
	P. ovale spp.	47	102	2,758	6,219	29,656	66,948
Extreme complexity	*Plasmodium* spp.	86		5,968		64,390	

Supplementary Table 15. Data underlying Figure 5c showing the impact of underestimated complexity on metrics that count diverse parasites. In this table, "Adjusted" represents adjusted estimates to account for deeper volume sampling (i.e. 100µL). Mean Pf-MOI and adjusted mean Pf-MOI estimated from ≥6-year-old individuals sampled from the larger DBS survey were scaled up to approximate the *P. falciparum* census population size in the Bongo DBS survey, Bongo District, and the Upper East Region of Ghana without age stratification.

Counting Diverse Parasites		**Survey in Bongo (1,455 individuals)**		**Scale up for Bongo District (102,004 individuals)**		**Scale up for Upper East Ghana (1,100,676 individuals)**	
		**DBS**	**Adjusted**	**DBS**	**Adjusted**	**DBS**	**Adjusted**
Mean MOI	*P. falciparum*	0.52	1.00				
P. falciparum census population size	*P. falciparum*	752	1,454	52,720	101,953	568,872	1,100,126
