# Supplementary material for: Metagenomic complexity of high, seasonal transmission of Plasmodium spp. in asymptomatic carriers in Northern Sahelian Ghana
Source: Commun Med (Lond). 2025 Sep 10;5:386. doi: 10.1038/s43856-025-01088-y (PMC12423318; doi:10.1038/s43856-025-01088-y)
Supplement: Supplementary file 2 — Reporting Summary [file 43856_2025_1088_MOESM2_ESM.pdf]

nature portfolio

Corresponding author(s): Mun Hua Tan

Last updated by author(s): Jun 12, 2025

Reporting Summary

Nature Portfolio wishes to improve the reproducibility of the work that we publish. This form provides structure for consistency and transparency in reporting. For further information on Nature Portfolio policies, see our Editorial Policies and the Editorial Policy Checklist.

Statistics

For all statistical analyses, confirm that the following items are present in the figure legend, table legend, main text, or Methods section.

n/a	Confirmed	
☐	☒	The exact sample size (n) for each experimental group/condition, given as a discrete number and unit of measurement
☐	☒	A statement on whether measurements were taken from distinct samples or whether the same sample was measured repeatedly
☐	☒	The statistical test(s) used AND whether they are one- or two-sided<br>*Only common tests should be described solely by name; describe more complex techniques in the Methods section.*
☐	☒	A description of all covariates tested
☐	☒	A description of any assumptions or corrections, such as tests of normality and adjustment for multiple comparisons
☐	☒	A full description of the statistical parameters including central tendency (e.g. means) or other basic estimates (e.g. regression coefficient) AND variation (e.g. standard deviation) or associated estimates of uncertainty (e.g. confidence intervals)
☐	☒	For null hypothesis testing, the test statistic (e.g. F, t, r) with confidence intervals, effect sizes, degrees of freedom and P value noted<br>*Give P values as exact values whenever suitable.*
☐	☒	For Bayesian analysis, information on the choice of priors and Markov chain Monte Carlo settings
☒	☐	For hierarchical and complex designs, identification of the appropriate level for tests and full reporting of outcomes
☐	☒	Estimates of effect sizes (e.g. Cohen's d, Pearson's r), indicating how they were calculated

Our web collection on statistics for biologists contains articles on many of the points above.

Software and code

Policy information about availability of computer code

Data collection	No software was used.
Data analysis	The following published software was used to analyse data:<br>- DBLaCleaner pipeline (v1.0)<br>- clusterDBLa pipeline (v1.0)<br>- classifyDBLa pipeline (v1.0)<br>- MOI estimation from varcoding data (https://github.com/qzhan321/Bayesian-formulation-varcoding-MOI-estimation)<br><br>Custom R code used to generate results and figures is available on GitHub (https://github.com/mh-tan/Metagenomic_Complexity_Plasmodium).

For manuscripts utilizing custom algorithms or software that are central to the research but not yet described in published literature, software must be made available to editors and reviewers. We strongly encourage code deposition in a community repository (e.g. GitHub). See the Nature Portfolio guidelines for submitting code & software for further information.

Data

Policy information about availability of data

All manuscripts must include a data availability statement. This statement should provide the following information, where applicable:

- Accession codes, unique identifiers, or web links for publicly available datasets
- A description of any restrictions on data availability
- For clinical datasets or third party data, please ensure that the statement adheres to our policy

P. falciparum DBLα tag sequences have been deposited to Genbank under the accession PRJNA1266761. Primer sequences, DBLα type sequences, and data tables underlying results are available on GitHub (https://github.com/mh-tan/Metagenomic_Complexity_Plasmodium). The individual age data are not publicly available due to ethical reasons. Requests for data on individual age classes corresponding to P. falciparum isolates should be made by contacting the Malaria Reservoir Study Team represented by Prof. Karen Day (karen.day@unimelb.edu.au; Response timeframe: ~1 month), in order to discuss how these data will be utilised for academic or research purposes and, if appropriate, to identify opportunities for collaboration.

Human research participants

Policy information about studies involving human research participants and Sex and Gender in Research.

Reporting on sex and gender	For this survey in November 2020, there were no exclusions based on sex or gender. Women that were pregnant on the day of the survey were not eligible for enrolment and were excluded. A detailed breakdown of this study population in sex and age categories is available in Tables S1 and S2.
Population characteristics	This information has been previously provided in Tiedje et al. AJTMH (2017), Tiedje, Oduro, et al. PLOS Global Public Health (2022), and Tiedje, Zhan, et al. eLife (2023), which are referenced in this work. We provide a summary as follows: All study participants enrolled in November 2020 were from Bongo District, Ghana and both male and female volunteers ≥ 6 years of age were sampled from four age groups (i.e., 6-10, 11-20, 21-39, and ≥ 40 years). This age range was selected so that we could investigate the P. falciparum reservoir that exists across all ages in areas of high seasonal malaria transmission, like Ghana. Children <5 years at the time the survey was conducted were receiving seasonal malaria chemoprevention (SMC) and were excluded. A detailed breakdown of this study population in age categories is available in Tables S1 and S2.
Recruitment	This information has been previously provided in Tiedje et al. AJTMH (2017), Tiedje, Oduro, et al. PLOS Global Public Health (2022), and Tiedje, Zhan, et al. eLife (2023), which are referenced in this work. We provide a summary as follows: During the survey in November 2020, the study team informed the participants by visiting their respective homes and inviting them to participate in the study. Individual informed consent was obtained in the local language from each enrolled participant by signature/thumbprint accompanied by the signature of an independent witness. A parent or guardian provided consent for children under the age of 18 years, and all children between the ages of 12 and 17 years also provided assent. Specifically for this study, supplementary consent was sought from 200 enrolled participants in Vea/Gowrie to collect ~5mL of whole blood. For this collection, 50 participants were randomly chosen from four age groups (i.e., 6-10, 11-20, 21-39, and ≥ 40 years). For each enrolled participant, a unique study ID (i.e. MRS####) was assigned, and these were linked with the consent/assent forms, structured questionnaires, and the blood samples taken during the study.
Ethics oversight	This information has been previously provided in Tiedje et al. AJTMH (2017), Tiedje, Oduro, et al. PLOS Global Public Health (2022), and Tiedje, Zhan, et al. eLife (2023), which are referenced in this work. We provide a summary as follows: This study was reviewed and approved by the ethics committees at the Navrongo Health Research Centre (Ghana) , The University of Melbourne (Australia), The University of Chicago (USA), and New York University (USA).

Note that full information on the approval of the study protocol must also be provided in the manuscript.

Field-specific reporting

Please select the one below that is the best fit for your research. If you are not sure, read the appropriate sections before making your selection.

☒ Life sciences ☐ Behavioural & social sciences ☐ Ecological, evolutionary & environmental sciences

For a reference copy of the document with all sections, see nature.com/documents/nr-reporting-summary-flat.pdf

Life sciences study design

All studies must disclose on these points even when the disclosure is negative.

Sample size	This information has been previously provided in Tiedje et al. AJTMH (2017), Tiedje, Oduro, et al. PLOS Global Public Health (2022), and Tiedje, Zhan, et al. eLife (2023), which are referenced in this work. We provide a summary as follows: In June 2012, an enumeration/demographic survey was completed in Bongo District, Ghana. This enumeration/demographic data was used to establish the villages, sections, and compounds in two catchment areas selected in Bongo District. Age-stratified sampling was completed based on these demographic data to reflect the underlying age characteristics of the population. At the time the study was designed in 2012, age-specific malaria prevalence data was not available for Bongo District. Consequently, malaria prevalence data from neighbouring Kassena-Nankana District, Ghana was used.

	Based on these data an estimated risk ratio of 3.0 during the dry season for malaria prevalence between the catchments areas was used to calculate the survey samples sizes. Therefore, at a 95% confidence level, 80% power and sample ratio of 1:1 between the catchment areas the estimated sample size per catchment area was 865, allowing for a 15% nonresponse rate. Based on these numbers, ~1,000 participants per catchment area were recruited with ~2,000 participants being enrolled during each survey. Data obtained from each participant sample represents an independent experiment.<br><br>From this population, 200 individuals were randomly chosen for this study. A power analysis based on the proportion of multiclonal infections (i.e. Pf-MOI > 1) in each pair of 1µL vs larger pRBC volume determined that there a sample size of 200 individuals was sufficient to achieve statistical power of 0.80 (α=0.05, alternative="two.sided") for comparison of different pRBC volumes when all individuals were considered. However, larger sample sizes per host age group are needed for age-stratified Pf-MOI corrections, as we note in the article.
Data exclusions	Of the 200 individuals, a total of 12 were excluded (Table S1). Nine were excluded as these could not be matched accurately to epidemiological data. The remaining three individuals were excluded as these were later confirmed to be symptomatic (i.e. febrile and microscopically-positive for P. falciparum). In terms of study design, pregnant women and children <5 years old were excluded, the latter group was receiving seasonal malaria chemoprevention (SMC).
Replication	Forty isolates were randomly selected as repeats to assess the reproducibility, representing ~20% of sample size. Analyses of outcomes between repeats showed high concordance of findings, supporting a robust approach in this study.
Randomization	For this study, 50 participants were chosen randomly for each of the four age groups (i.e., 6-10, 11-20, 21-39, and ≥ 40 years), whilst ensuring female and male participants are represented almost equally.
Blinding	Blinding was not suitable for the analyses performed as this was an observational cross-sectional study. Although the whole population in Bongo District had undergone sequential malaria control interventions, including long-lasting insecticidal nets (LLINs), three rounds of indoor residual spraying (IRS) (2013-2015), and five consecutive years (2016-2020) of seasonal malaria chemoprevention (SMC), we did not assign participants to interventions.

Reporting for specific materials, systems and methods

We require information from authors about some types of materials, experimental systems and methods used in many studies. Here, indicate whether each material, system or method listed is relevant to your study. If you are not sure if a list item applies to your research, read the appropriate section before selecting a response.

Materials & experimental systems

n/a	Involved in the study	
☒	☐	Antibodies
☒	☐	Eukaryotic cell lines
☒	☐	Palaeontology and archaeology
☒	☐	Animals and other organisms
☒	☐	Clinical data
☒	☐	Dual use research of concern

Methods

n/a	Involved in the study	
☒	☐	ChIP-seq
☒	☐	Flow cytometry
☒	☐	MRI-based neuroimaging
